# Bacteriophage Sf6 Tailspike Protein for Detection of *Shigella flexneri* Pathogens

**DOI:** 10.3390/v10080431

**Published:** 2018-08-15

**Authors:** Sonja Kunstmann, Tom Scheidt, Saskia Buchwald, Alexandra Helm, Laurence A. Mulard, Angelika Fruth, Stefanie Barbirz

**Affiliations:** 1 Physical Biochemistry, University of Potsdam, 14476 Potsdam, Germany; sonja.kunstmann@mpikg.mpg.de (S.K.); ts599@cam.ac.uk (T.S.); sb165329@uni-greifswald.de (S.B.); ahelm@uni-potsdam.de (A.H.); 2 Institut Pasteur, Unité de Chimie des Biomolécules, 28 rue du Roux, 75015 Paris, France; laurence.mulard@pasteur.fr; 3 CNRS UMR 3523, Institut Pasteur, 75015 Paris, France; 4 National Reference Centre for Salmonella and other Bacterial Enterics, Robert Koch Institute, 38855 Wernigerode, Germany; frutha@rki.de

**Keywords:** *Shigella flexneri*, bacteriophage, tailspike proteins, O-antigen, serotyping, microtiter plate assay, fluorescence sensor

## Abstract

Bacteriophage research is gaining more importance due to increasing antibiotic resistance. However, for treatment with bacteriophages, diagnostics have to be improved. Bacteriophages carry adhesion proteins, which bind to the bacterial cell surface, for example tailspike proteins (TSP) for specific recognition of bacterial O-antigen polysaccharide. TSP are highly stable proteins and thus might be suitable components for the integration into diagnostic tools. We used the TSP of bacteriophage Sf6 to establish two applications for detecting *Shigella flexneri* (*S. flexneri*), a highly contagious pathogen causing dysentery. We found that Sf6TSP not only bound O-antigen of *S. flexneri* serotype Y, but also the glucosylated O-antigen of serotype 2a. Moreover, mass spectrometry glycan analyses showed that Sf6TSP tolerated various O-acetyl modifications on these O-antigens. We established a microtiter plate-based ELISA like tailspike adsorption assay (ELITA) using a Strep-tag^®^II modified Sf6TSP. As sensitive screening alternative we produced a fluorescently labeled Sf6TSP via coupling to an environment sensitive dye. Binding of this probe to the *S. flexneri* O-antigen Y elicited a fluorescence intensity increase of 80% with an emission maximum in the visible light range. The Sf6TSP probes thus offer a promising route to a highly specific and sensitive bacteriophage TSP-based *Shigella* detection system.

## 1. Introduction

*Shigella* spp. cause gastrointestinal disease, ranging from mild diarrheal episodes to complicated dysentery. Approximately 65,000 of the 190 million cases reported worldwide each year are fatal, mostly of children under five years [1,2]. Thereby, most infections in developing countries are caused by *Shigella flexneri* (*S. flexneri*) strains, with serotype 2a as the most prominent [3]. Although humans are the primary reservoir of *Shigella* spp. [4], an increasing number of resistant *Shigella* strains are found [5], with livestock farming as a potential source [6]. These emerging resistances against antimicrobial agents force the community not only to find new drugs but also to keep outbreaks and contaminations to a minimum by fast and reliable diagnostic tools. *Shigella* spp. and closely related entero-invasive *E. coli* (EIEC) acquired a large virulence plasmid (pINV) as they evolved from ancestral *E. coli* [7,8]. It is therefore a demanding task to specifically distinguish these strains especially in the presence of other, non-invasive *E. coli* strains [9].

A number of certified protocols describe the detection of Gram-negative pathogens with antibodies as well as with classic selective microbiological media, also in combination with Real-Time PCR [10]. The time frame of these methods, however is often limited by the bacterial enrichment procedures to increase cell numbers to the limits of detection of any given method. A suitable sensor hence should give a rapid signal even at low concentrations. For example, as little as ten colony forming units (cfu) per milliliter of *S. flexneri* can cause an infection in the host [11]. In complement to the ongoing development of immunochromatographic dipsticks for use at the patient’s bedside [12], bacteriophage-based devices are promising alternatives for pathogen detection at low cfu numbers. Here, either complete engineered luminescent phage particles or isolated cell-wall binding proteins have been employed to identify concentrations of 10^2^ to 10^3^ cfu/mL; also, a *Shigella* specific T4-like reporter phage has been employed lately [13,14,15,16,17]. Recent shigellosis outbreaks also stimulated the isolation of 16 new phages specific for *Shigella* spp. [18]. Tailed bacteriophages contain a variety of proteins to mediate initial cell wall contacts [19,20,21]. In O-antigen specific bacteriophages, tailspike proteins (TSP) are essential mediators for host-cell adsorption and infection initiation [22]. They bind and enzymatically cleave O-polysaccharides of Gram-negative bacteria with high specificity [23]. This high specificity of TSP towards bacterial cell wall glycan structures together with their high stability makes them promising candidates for diagnostic applications [24,25,26]. Moreover, especially in *Shigella* spp., the O-antigen has been described as an important virulence factor [27,28] that might be addressed by a specific TSP-based probe.

**Scheme 1:**
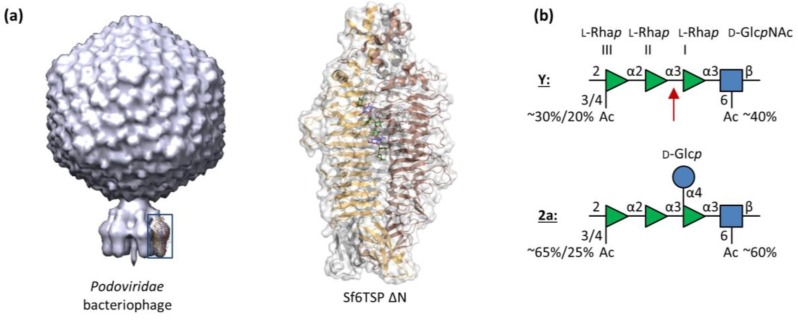
(**a**) Sf6 tailspike (TSP) is located in the non-contractile podovirus tail (framed in blue, EMDB: 1222). The trimeric β-helix structure of the Sf6TSP without capsid head binding domain (ΔN) crystallized with an octasaccharide (sticks, Rha*p* = green, Glc*p*NAc = blue) is shown as cartoon in gray, brown and yellow for each subunit (PDB ID: 4URR) [30,40]. (**b**) *S. flexneri* O-antigen repeat units of serotypes Y and 2a are depicted according to the symbolic nomenclature for glycans (SNFG) [41]. Serotypes Y and 2a can include non-stoichiometric O-acetylation at Rha*p*III-O3/4 or Glc*p*NAc-O6 [36,42]. The cleavage site of the Sf6TSP endorhamnosidase is indicated by the red arrow.

The tailspike protein of the temperate *S. flexneri* podovirus Sf6 (Sf6TSP) binds the *S. flexneri* serotype Y polysaccharide (*gp14*) (Scheme 1a) [29,30,31,32,33,34,35]. This high specificity makes Sf6TSP suitable to be used as a sensor for selective *S. flexneri* strain detection. The structures of *S. flexneri* O-antigen repeat units (RUs) have serotype specific glucosylation and O-acetylation patterns on a rhamnose-rich backbone structure [36,37,38]. Sf6TSP has endorhamnosidase activity and cleaves α-(1→3) linkages between two rhamnoses. Main hydrolysis products of serotype Y O-polysaccharides are octasaccharides consisting of two RUs with the structure: [→3)-α-l-Rha*p*I-(1→3)-β-d-Glc*p*NAc-(1→2)-α-l-Rha*p*III-(1→2)-α-l-Rha*p*II-(1→] (Scheme 1b) [33]. Bacteriophage Sf6 has been described to infect *S. flexneri* with serotype Y and X but was unable to use serotype 2a cells as a host [39]. In the present work, we have exploited the capacity of using Sf6TSP as a *S. flexneri*-sensitive probe. We show that engineered Sf6TSP constructs can be applied in a rapid microtiter plate-based assay or as sensitive fluorescent probes for *S. flexneri* detection. Moreover, we analyzed the specificity range of the Sf6TSP probe and found that it was also able to recognize *S. flexneri* serotype 2a O-antigen.

## 2. Materials and Methods

### 2.1. Materials and Bacterial Strains

Phosphate buffered saline (PBS): 16 mM Na_2_HPO_4_, 4 mM KH_2_PO_4_, 115 mM NaCl, pH 7.6. TE-buffer: 50 mM Tris/HCl, 5 mM ethylenediaminetetraacetic acid (EDTA), pH 7.6. PBS-T: PBS buffer supplemented with additional 200 mM NaCl (final conc. 315 mM NaCl) and 0.2% (*v*/*v*) Tween20. IANBD: 2-iodo-*N*-methyl-*N*-[2-[methyl(7-nitro-2,1,3-benzoxadiazol-4-yl)amino]ethyl] acetamide (Invitrogen, Thermo Fisher Scientific GmbH, Dreieich, Germany). All chemicals were of analytical grade, and ultrapure water was used throughout. *Shigella* strains were characterized and provided by the National Reference Centre for *Salmonella* and other Bacterial Enterics in Wernigerode, Germany (Wernigerode collection) [43]. While *S. flexneri* 2a O-antigen icosasaccharide fragments of the structure [→2)-α-l-Rha*p*III-(1→2)-α-l-Rha*p*II-(1→3)-[α-d-Glc*p*-(1→4)]-α-l-Rha*p*I-(1→3)-β-d-Glc*p*NAc-(1→]_4_-O(CH_2_)_2_NH_2_ was synthesized as previously published [44]. Lipopolysaccharide (LPS) of *S. flexneri* Y and 2a was a gift from Nils Carlin (Scandinavia Biopharma, Solna, Sweden). Sera for O-serotyping were purchased from Sifin GmbH (Berlin, Germany). Cloning and purification of Sf6TSP*wt* (wild type) and of the hydrolysis deficient mutant Sf6TSP E366A/D399A have been described [29,30].

### 2.2. Cloning and Protein Purification

An N-terminal His_6_-tag and TEV protease cleavage site were introduced by amplification PCR based on described plasmids [29,30]. All cysteine mutants were derived using the QuickChange kit (Agilent, Santa Clara, CA, USA). To add an N-terminal Strep-tag^®^II (IBA, Göttingen, Germany), Sf6TSP E366A/D399A genes were excised with restriction enzymes *Sfo*I and *EcoR*I after introduction of a *Sfo*I cleavage site by single point mutagenesis PCR and the fragment was ligated into the plasmid pPR-IBA102 (IBA, Göttingen, Germany) and sequenced (GATC Biotech AG, Konstanz, Germany).

Sf6TSP E366A/D399A and cysteine mutants were obtained from heterologous expression of the corresponding plasmids in *E. coli* BL21(DE3) as described [29]. Proteins were purified via Ni-chelating affinity chromatography and the His_6_-tag was proteolytically removed by TEV protease. Purification of StrepII-Sf6TSP and Sf6TSP*wt* has been described elsewhere [24,29].

### 2.3. Oligo- and Polysaccharide Preparation

Detoxified LPS consisting of O-antigen and core (polysaccharide) from natural *S. flexneri* isolates was purified as described [45,46]. Briefly, bacteria were grown in 10 L LB medium overnight at 37 °C. Bacteria pellets were washed twice with 10 mM Tris/HCl, 2 mM MgCl_2_ pH 7.6 and twice with water before suspension in 100 mL 10% acetic acid. Bacteria were hydrolyzed twice for 1.5 h at 99 °C. Collected supernatants were adjusted to pH 7.0 and the polysaccharide was precipitated in 80% ethanol at −40 °C for 2 h. Pellets were solubilized in 10 mM Tris/HCl, 4 mM MgCl_2_ pH 7.8 and treated with 2 U μL^−1^ benzonase for 3 h at 37 °C and 15 μg mL^−1^ proteinase K for 3 h at 65 °C and purified by ethanol precipitation and treatment with DEAE sepharose. Purity of polysaccharide samples was assumed if A_260_ and A_280_ were beyond 0.1 for a 1 mg mL^−1^ solution. Polysaccharide cleavage by TSP and capillary electrophoresis laser induced fluorescence (CE-LIF) of the reaction products have been described [30].

Oligosaccharides were prepared as described [45,46]. For this purpose, *S. flexneri* polysaccharide (38 mg mL^−1^) was incubated with 50 μg mL^−1^ Sf6TSP*wt* overnight at room temperature (RT) and freeze dried. Mixtures were purified via size exclusion chromatography on a HiLoad 26/60 Superdex 30 column (GE Healthcare, München, Germany) with water as the mobile phase. Oligosaccharide elution was monitored with a refractive index detector and their purity assessed with matrix-assisted laser-desorption ionization mass spectrometry (MALDI-TOF MS) as described elsewhere [24,30].

### 2.4. ELISA Like Tailspike Adsorption (ELITA) Assay

The ELITA principle and set up have been described [24]. Briefly, bacteria were grown in 10 mL LB medium until OD_600_ = 0.7–1.0. Bacteria were harvested and inactivated with 2% glutaraldehyde for 1 h. After two wash steps PBS, 200 μL bacterial suspensions in PBS at OD_600_ = 0.1 were adsorbed overnight at 4 °C to a 96-well microtiter plate (Nunc F Maxisorp, Thermo Fisher Scientific GmbH, Dreieich, Germany). Adsorbed bacteria were washed twice with 210 μL PBS. Each well was blocked with 220 μL 1% BSA in PBS for 2 h at RT and washed once with 220 μL PBS. Each well was incubated with 190 μL 30–80 nM StrepII-Sf6TSP for 20 min under shaking. Four wash steps with 200 μL PBS-T removed unbound TSP. For detection, each well was incubated with 190 μL HRP-*Strep*-Tactin^®^ conjugate (IBA, Göttingen, Germany) (1:10,000 in PBS-T) for 15 min at 130 rpm. Unbound conjugate was removed by three washings steps with 200 μL PBS-T and one washing step with 210 μL PBS. Binding was quantified by adding 200 μL 1 mg mL^−1^ O-phenylenediamine with 0.03% H_2_O_2_ (*v*/*v*) in 50 mM citrate buffer pH 6.0 for 5 min and absorption was analyzed at 492 nm after stopping the reactions with 50 μL 2 M H_2_SO_4_.

### 2.5. Fluorescence Labeling

Cysteine mutants of Sf6TSP E366A/D399A (1 mg mL^−1^) were treated with 20 mM Tris(2-carboxyethyl)phosphine (TCEP) in 400 mM sodium phosphate pH 7 at 4 °C overnight and the reducing agent was removed by ultrafiltration. Reduced protein samples (1 mg mL^−1^ in 50 mM sodium phosphate pH 7) were fluorescently labeled in the dark with 10 mM IANBD in 20% (*v*/*v*) DMSO for 1 h at 56 °C. Free dye was removed by size exclusion chromatography on a PD10 column and protected from light. Purity of labeled protein solutions was determined by size exclusion chromatography with a Superdex 200 10/300 column with fluorescence detection (λ_ex_ = 492 nm, λ_em_ = 545 nm). Labeling efficiency was calculated as described elsewhere: C_f_/C_p_ = (A_478_·ε_280-TSP_)/((A_280_·ε_478-IANBD_) − (A_478_·ε_280-IANBD_)) with ε_280-TSP_ = 54,320 M^−1^cm^−1^, ε_478-IANBD_ = 50,300 M^−1^cm^−1^ and ε_280-IANBD_ = 1745 M^−1^cm^−1^ [47].

### 2.6. Fluorescence Spectroscopy

Intrinsic protein fluorescence was excited at 280 nm and emission was monitored between 300 and 450 nm as described [30]. The *N*-methyl-*N*-[2-[methyl(7-nitro-2,1,3-benzoxadiazol-4-yl)amino]ethyl] (NBD) label was excited at 492 nm and emission spectra were monitored between 500 and 650 nm. Spectra were recorded 3 min after addition of oligo- or polysaccharides and dilution-corrected. Binding constants were obtained from fitting the data to the binding isotherms obtained from amplitude changes at 340 nm (protein) and 540 nm (NBD), respectively, as described elsewhere [48].

### 2.7. Surface Plasmon Resonance

The experiment was performed as described previously [45]. Briefly, immobilization and interaction measurements were recorded at a BIAcore J instrument (GE Healthcare Europe GmbH, Freiburg, Germany) with filtered (0.45 μm pore size) and degassed buffers at 25 °C. Flow rates in the BIAcore J instrument are not further quantified than low, medium, and fast by the instrument instructions. Immobilization and measurements were performed at a low and a medium flow rate, respectively. For immobilization, the carboxymethyldextran surface chip CMD200d (Xantec, Düsseldorf, Germany) was activated with 0.05 M NHS (N-hydroxysuccinimide) and 0.05 M EDC (1-Ethyl-3-(3-dimethylaminopropyl)carbodiimide) in 50 mM MES pH 5.0 (2-(*N*-morpholino)ethanesulfonic acid) for 6 min. Protein samples (182 µM) were immobilized in 20 mM sodium acetate buffer pH 4 by five consecutive injections for 6 min each. Residual reactive groups on the chip surface were inactivated with a 6 min injection of 1 M ethanolamine pH 8. The reference channel was immobilized with P22TSPΔN accordingly. Interaction experiments were performed in 10 mM sodium phosphate buffer pH 7 with oligosaccharide concentrations between 0–800 µM for 60 s. Response unit maxima were recorded 2 s before the end of the injection.

## 3. Results

### 3.1. Sf6TSP Binds and Enzymatically Cleaves the S. flexneri Serotype 2a O-Antigen

**Figure 1:**
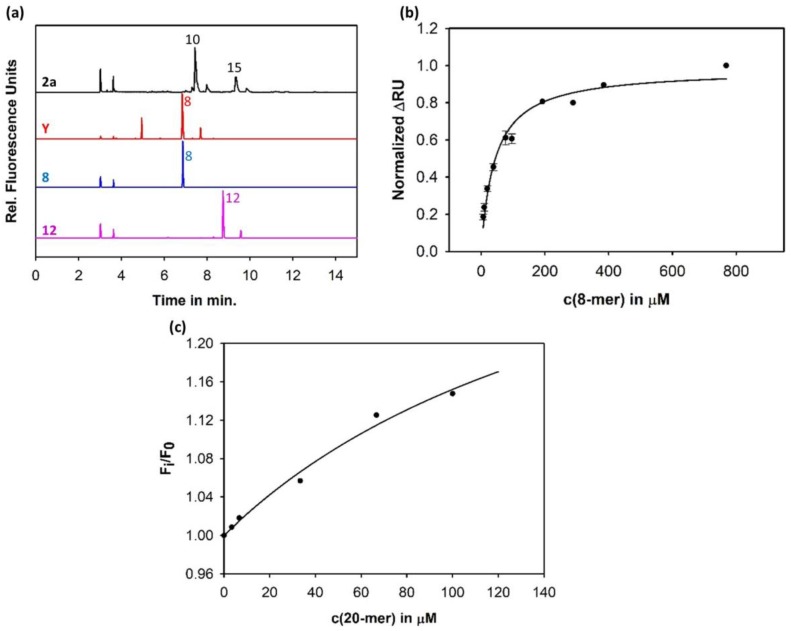
(**a**) Capillary electrophoresis laser induced fluorescence CE-LIF analysis of oligosaccharide products obtained by Sf6TSP cleavage from *S. flexneri* lipopolysaccharide (LPS) preparations of serotype Y (red) or 2a (black). Purified serotype Y dodecasaccharide (violet) or octasaccharide (blue) standards are also shown and numbers indicate the size of the oligosaccharide fragments. (**b**) Normalized surface plasmon resonance signals of *S. flexneri* oligosaccharides binding to surface-immobilized Sf6TSP E366A/D399A at 25 °C. The solid line represents the isotherm obtained from the fit to a single independent binding site model and yielded a *K_D_* of 42.81 ± 6.69 μM for the octasaccharide ligand. Standard deviations from three independent measurements are indicated at selected concentrations. (**c**) Increase of Sf6TSP E366A/D399A intrinsic protein fluorescence upon addition of a synthetic *S. flexneri* serotype 2a fragment: [→2)-α-l-Rha*p*III-(1→2)-α-l-Rha*p*II-(1→3)-[α-d-Glc*p*-(1→4)]-α-l-Rha*p*I-(1→3)-β-d-Glc*p*NAc-(1→]_4_-O(CH_2_)_2_NH_2_ in 50 mM sodium phosphate buffer pH 7.0 and 184 nM Sf6TSP. Fitting of the data to a single independent site binding isotherm resulted in a dissociation constant *K_D_* of 186 ± 91 μM.

The Sf6TSP binding site has been well characterized to interact with O-antigen fragments of serotype Y that lack branching glucose modifications (*cf.* Scheme 1) [16]. Accordingly, phage Sf6 can lyse *S. flexneri* serotype Y strains. However, although *S. flexneri* 2a strains were found insensitive for Sf6 lysis, they showed a weak adsorption of Sf6 phage particles [32]. Thus, we purified *S. flexneri* 2a lipopolysaccharide (LPS), incubated it with Sf6TSP*wt*, and analyzed the products with capillary electrophoresis (Figure 1a). Sf6TSP*wt* released oligosaccharides from serotype 2a O-antigen that had shifted retention times when compared with octa- and dodecasaccharide obtained from serotype Y.

It was shown before that Sf6TSP could release tetrasaccharides from serogroup Y oligosaccharides and polysaccharides [29,30]. However, in the present analysis we did not observe tetrasaccharide products in digests of 2a LPS with Sf6TSP*wt* (Figure 1a).

We used the enzymatically inactive Sf6TSP variant E366A/D399A to compare binding affinities of the different O-serogroup oligosaccharides. From binding isotherms obtained from surface plasmon resonance spectroscopy (SPR) on immobilized Sf6TSP we calculated a dissociation constant *K_D_* of 43 ± 7 µM for serogroup Y octasaccharides (Figure 1b). However, the amount of sample consumption prevented further measurements with longer fragments of other serotypes with SPR. Intrinsic protein fluorescence here can be alternatively employed, given that binding elicits a detectable signal change [45]. Whereas serogroup Y octasaccharides showed no influence on intrinsic protein fluorescence, a fully synthetic serotype 2a O-antigen fragment containing four repeat units notably increased the intrinsic fluorescence of the enzymatically inactive Sf6TSP variant E366A/D399A (*K_D_* = 186 ± 91 µM; Figure 1c). The Sf6TSP oligosaccharide binding site thus tolerated an α-(1→4) glucosyl modification at Rha*p*I as present in serotype 2a, although at reduced affinity when compared to serotype Y ligands.

### 3.2. Binding of Sf6TSP to S. flexneri O-Antigens with O-Acetyl and Glucosyl Modifications

Characterization of *S. flexneri* from isolates of notifiable dysentery cases usually encompasses slide agglutination tests with monoclonal antibodies in either monospecific or polyspecific reagent mixtures. Moreover, the O-antigen gene polymerase *wzx* serves as genetic marker for a PCR-based analysis [49]. However, these methods do not always return consistent results for unambiguous identification of *S. flexneri*. We obtained four *S. flexneri* Y and 2a isolates of the Wernigerode collection (Table 1). One of these strains had been designated as *S. flexneri* 2a by slide agglutination but was negative in the PCR test.

**Figure 2:**
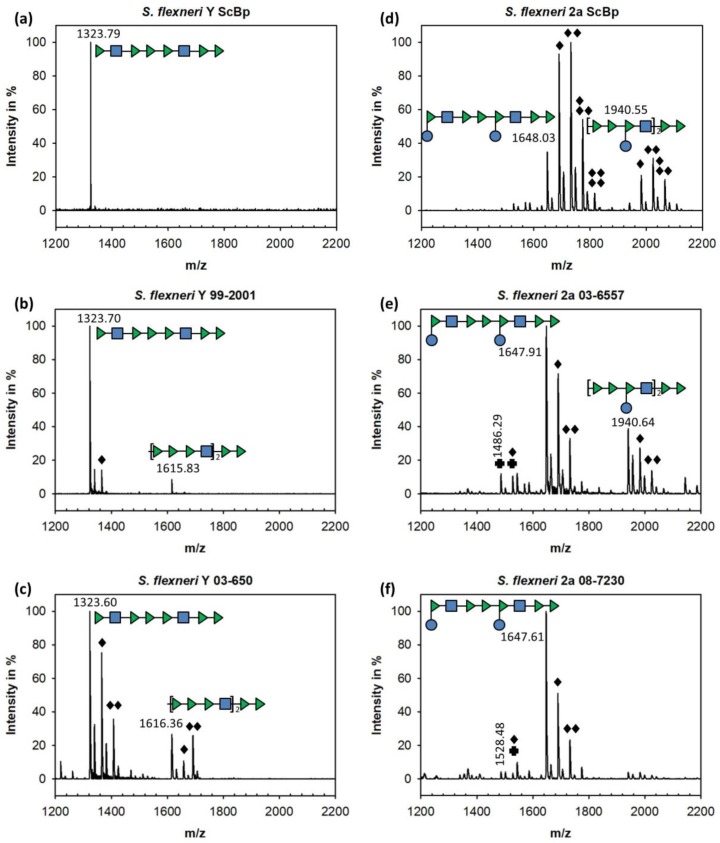
MALDI-TOF MS analysis of oligosaccharides enzymatically released from *S. flexneri* O-antigen (overnight at room temperature) by Sf6TSP*wt* and isolated by size exclusion chromatography (see Figures S1–S6 and Tables S1–S6). (**a**,**d**) *S. flexneri* Y and 2a probes from ScBp. (**b**,**c**,**e**,**f**) *S. flexneri* isolates from the Wernigerode collection. Oligosaccharide structures are given with monosaccharides in SNFG representation [41] (Rha*p*: green triangle, Glc*p*NAc: blue square, Glc*p*: blue circle). Modifications by O-acetylation (black diamonds), and monoglucosylated products (black crosses) are indicated.

To further characterize the composition of O-antigen structures found in the four *S. flexneri* isolates, we prepared the polysaccharide and incubated it with Sf6TSP*wt* to obtain oligosaccharides. Moreover, we used two serotype Y and 2a lipopolysaccharides isolated at Scandinavia Biopharma (ScBp). Cleavage products were separated by size exclusion chromatography (Figures S1–S6) and their composition was analyzed by MALDI-TOF mass spectrometry (MALDI-MS) (Figure 2 and Tables S1–S6). Oligosaccharides composed of up to seven repeating units could be identified (Figure S6, Table S6), with octa- and decasaccharides as the main products for serotype Y and 2a, respectively. Depending on the *S. flexneri* strain analyzed, we found different oligosaccharide O-acetylation patterns. Strains with a larger variety of modifications also showed more heterogeneous oligosaccharide mixtures in size exclusion chromatography. Y strain 99–2001 showed mainly non-O-acetylated octasaccharide and about 10% mono-O-acetylated species whereas in Y strain 03–650 about half of the octasaccharide was mono- or di-O-acetylated (Figure 2a–c). Our MALDI-MS analysis set-up could not identify the positions of O-acetylations in the respective oligosaccharides, but NMR studies had shown before that Rha*p*III of serotype Y strains was acetylated at O3 to 30% and at O4 to 20%; additionally, 40% of Glc*p*NAc-O6 were acetylated [36]. Moreover, the degree of O-acetylation in these positions was clearly increased in *S. flexneri* serotype 2a O-antigens.

The 2a strains used in this work also had O-acetylated species. However, these *S. flexneri* 2a isolates showed smaller fractions of O-acetylated oligosaccharides compared to the 2a ScBp (Figure 2d). For the latter, we observed a complex distribution of oligosaccharide compounds after Sf6TSP*wt* cleavage, with for example up to four O-acetylations found in a decasaccharide. In contrast, oligosaccharides released from the 2a strains in this work contained high amounts of non-O-acetylated decasaccharides (Figure 2e–f). Moreover, minor peaks were detected with masses corresponding to oligosaccharides that lacked one glucose moiety resulting from the glucosylation process of the undecaprenyl pyrophosphate-O-antigen conjugate as non-glucosylated first repeating unit, what provides a measure for the amount of O-antigen chains [50,51]. Thus, oligosaccharide release analysis from strains with serotype Y and 2a O-antigens confirmed that Sf6TSP enzymatic digest is allowed for different degrees of O-acetylation, as naturally found in strains encountered in the field. Moreover, Sf6TSP could enzymatically cleave the *S. flexneri* 2a O-antigen, i.e., it tolerated a modification of the general serotype Y backbone by an α-(1→4) glucosylation at Rha*p*I.

### 3.3. ELITA: ELISA Like Tailspike Adsorption Assay for Detection of S. flexneri

**Figure 3:**
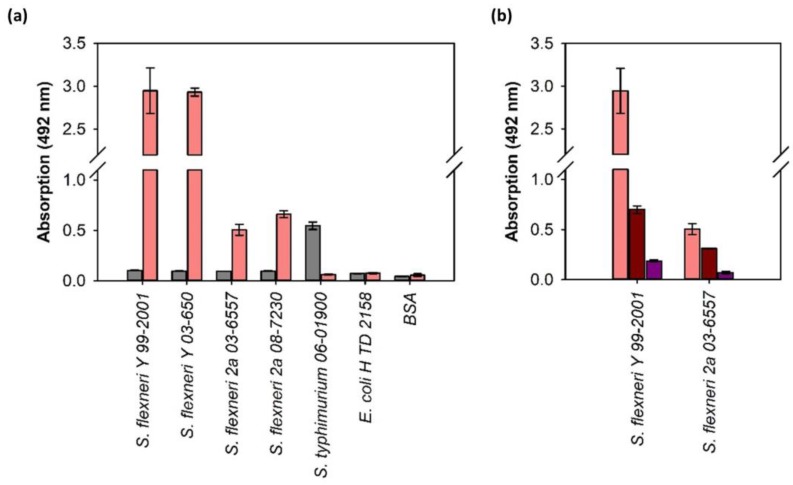
ELITA (ELISA-like tailspike adsorption assay). (**a**) Strep-tagged TSP probes of StrepII-Sf6 (pink) and StrepII-P22 (gray) were used for analysis of the indicated *Shigella* and *Salmonella* strains. *E. coli* and 1 mg/mL BSA were used as a negative control for both proteins. (**b**) To control the O-antigen specificity of the detection process, enzymatically active Sf6TSP*wt* (30 nM) was added to immobilized bacteria (dark red) before detection with StrepII-Sf6TSP. To compete with bacteria-associated O-antigen, an *S. flexneri* Y O-polysaccharide preparation (50 µg mL^−1^ *Sf*Y PS) was co-incubated with the StrepII-Sf6TSP (violet). For comparison, pink bars reproduced from (a) show StrepII-Sf6TSP detection of *S. flexneri* Y and 2a without further treatment (pink). Error bars show standard deviations from three sample replicates.

The specificity of Sf6TSP therefore suggested applying the protein as probe in an ELISA-like tailspike adsorption assay (ELITA) for rapid screening of bacteria in a microtiter plate-based format [24]. To apply Sf6TSP as specific *S. flexneri* probe in ELITA, we cloned an enzymatically inactive Sf6TSP E366A/D399A mutant carrying a N-terminal Strep-tag^®^II (StrepII-Sf6TSP). In ELITA, bacteria are grown to stationary phase, surface-adsorbed and serotype specifically detected with the N-terminally Strep-tag^®^II-modified tailspike proteins that can be quantified via Strep-Tactin^®^ labeled horseradish peroxidase [24]. The ELITA with Sf6TSP as a probe unambiguously identified all four isolates as *S. flexneri* strains, while control strains of *E. coli* and *Salmonella* Typhimurium (*S*. Typhimurium) did not elicit a signal (Figure 3a) [24]. The ELITA was also positive on a bacterial isolate that had been designated as *S. flexneri* 2a by slide agglutination, but was negative in the PCR test (Table 1). Signal intensities were about six times higher for *S. flexneri* Y strains compared to the 2a strains. The latter showed comparable ELITA signal intensities with a P22TSP control that bound *S*. Typhimurium [24]. Accordingly, the *Salmonella*-specific P22TSP exhibited baseline values against all four *Shigella* isolates tested. To confirm that detection was solely due to specific binding between Sf6TSP and the *S. flexneri* O-antigen, bacteria were incubated with enzymatically active Sf6TSP*wt* to remove bacterial surface O-antigen chains. This reduced the ELITA signals to 62% on Y and to 24% on 2a strains (Figure 3b). As a further control, we added a serotype Y polysaccharide preparation to the test. In this set-up, O-antigen binding sites on the bacterial surface competed with the free O-antigen polysaccharide in solution. Consequently, we now found a loss of signal because the Sf6TSP probe was quenched by the free polysaccharide and could no longer bind to the bacteria. Both controls are important to state that ELITA monitors an exclusively O-antigen specific process and that no unspecific binding of the TSP to other parts of the immobilized bacteria occurs. Hence, the ELITA protocol, initially established for *Salmonella* spp. detection, was successfully adapted as diagnostic tool to specifically identify *S. flexneri* Y and 2a species with the bacteriophage Sf6TSP probe.

### 3.4. Construction of an Sf6TSP Probe with O-Antigen Specific Fluorescence Amplitude Increase

As an alternative to a microtiter plate-based assay we wanted to exploit Sf6TSP’s high sensitivity for identification of *S. flexneri* with fluorescence detection. We chose the environment-sensitive *N*-methyl-*N*-[2-[methyl(7-nitro-2,1,3-benzoxadiazol-4-yl)amino]ethyl] (NBD) dye label to specifically elicit a signal only upon O-antigen binding. With NBD covalently linked to Sf6TSP binding of *S. flexneri*, O-antigen should then elicit an increased fluorescence emission signal in the visible spectrum due to a higher hydrophobicity around the conjugated label [52]. To couple NBD to Sf6TSP we introduced six-single-point cysteine mutations in and around the octasaccharide binding site of an enzymatically inactive Sf6TSP E366A/D399A variant (Table 2).

**Figure 4:**
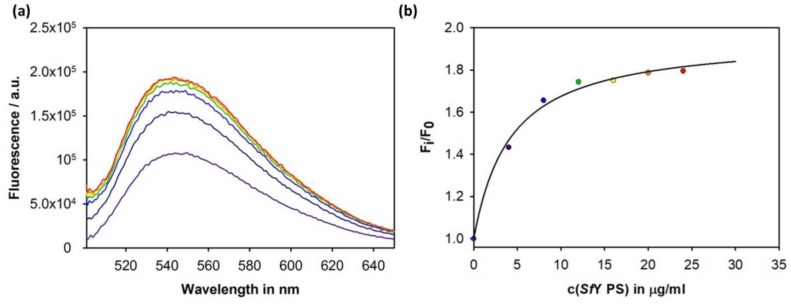
(**a**) Fluorescence emission spectra at 10 °C of Sf6TSP (184 nM) covalently linked to a *N*-methyl-*N*-[2-[methyl(7-nitro-2,1,3-benzoxadiazol-4-yl)amino]ethyl] (NBD) dye label in the absence (purple) or presence of increasing amounts of *S. flexneri* serotype Y polysaccharide preparations at λ_ex_ = 492 nm (Polysaccharide concentrations in µg mL^−1^: purple = 0; dark blue = 4; light blue = 8; green = 12; yellow = 16; orange = 20; and red = 24). (**b**) Binding isotherm of polysaccharide binding to Sf6TSP-NBD. Data was fitted to a single binding site model to obtain a polysaccharide half-saturation concentration of 4.1 µg mL^−1^ (Color of data points correspond to curves in (**a**)).

The high stability of Sf6TSP enabled a subsequent fluorescent labelling step at elevated temperatures (56 °C) and in the presence of 20% DMSO (Figure S7). Under these conditions, labelling efficiencies of approximately 20 to 60% were reached. Fluorescence amplitude increases at 540 nm were recorded upon addition of purified O-antigen preparations of *S. flexneri* serotype Y for the NBD labels placed at the different positions around the binding site. When NBD was coupled to a cysteine replacing for residue N340, the fluorescence intensity increased by about 80% upon polysaccharide binding (Table 2 and Figure 4a). Half saturation of the signal was reached at about 4 µg mL^−1^ in the presence of 184 nM labelled protein (Figure 4b). The NBD-labelled mutant S246C also showed an intensity increase of about 50%, whereas the other positions tested either showed low signals or were completely insensitive for O-antigen binding (Table 2). Furthermore, for the NBD-labelled Sf6TSP E366A/D399A N340C (Sf6TSP-NBD) we analyzed polysaccharide dissociation rates (Figure S8). The resulting kinetic relaxation traces of Sf6TSP-NBD upon polysaccharide binding did not change compared to those of the unlabeled protein described earlier [30]. This confirmed that polysaccharide binding was not essentially affected by the fluorescent label and the introduction of the cysteine mutations. In conclusion, we state that Sf6TSP-NBD is an efficient O-antigen detecting probe as the protein combines good labelling efficiencies with a strong increase of the fluorescence amplitude.

## 4. Discussion

In this work we have explored the possibilities for using Sf6TSP, the receptor binding protein of bacteriophage Sf6, as a sensor for *S. flexneri*. For this, we have analyzed the specificity of Sf6TSP towards the two *S. flexneri* serotypes Y and 2a. Moreover, we established two types of diagnostic read-out via an ELITA assay on microtiter plates or using a fluorescent probe sensitive for O-antigen binding.

### 4.1. Binding Specificity of the Sf6TSP Endorhamnosidase

O-antigen specific bacteriophages using TSP for receptor binding encounter cell surface glycans with highly variable structures, the latter depending on factors like other prophages or bacterial phase variations [24,53,54]. This leads to a continuous diversification of O-antigen structures, which is especially well illustrated when regarding the very diverse glucosylation and O-acetylation patterns of *S. flexneri* surface polysaccharides [36]. In O-antigen specific phages, TSP adsorption and enzymatic cleavage of the O-polysaccharide is a strictly necessary step for infection and can lead to DNA ejection *in vitro*, although the subsequent steps leading to particle opening are not yet understood to molecular detail [22]. The temperate bacteriophage Sf6 infects *S. flexneri* with serotypes X and Y but failed to infect all other serotypes including 2a [32,33,39]. O-antigen binding is necessary for Sf6 to infect its host, however, Sf6 also infected a related *E. coli* K-12 strain when this strain was mutated to express a serotype Y O-antigen [32]. In the present study however, we could show that Sf6TSP bound and cleaved all three *S. flexneri* serotype 2a O-antigens analyzed. The 2a O-antigen contains an additional α-(1→4)-linked glucose branch on Rha*p*I compared to the Y O-antigen (*cf.* Scheme 1). This modification most likely does not hamper the Sf6TSP O-antigen binding mode found for serotype Y octasaccharide. In the corresponding serotype 2a decasaccharide the additional glucose could be accommodated in the binding site because it would point away from the protein surface into the solvent [30]. Hence, in serotype 2a strains other processes necessary for phage injection are hampered besides O-antigen recognition and cleavage.

For bacteriophage Sf6, additional recognition of OmpA and OmpC protein receptors has been described, however, also *ompA-ompC* deletion strains can still be infected by Sf6 although with reduced efficiency [55]. Moreover, extracellular loops 2 and 4 of OmpA have been proposed to determine the ability of Sf6 to overcome the extracellular membrane [56]. It has also been found that the *Shigella* O-antigen polyrhamnose backbone is highly flexible and that glucosylation-related conformational changes can alter the presentation of proteins in the membrane [28,57]. As a consequence, certain O-antigens might have conformations that hamper phage particle access to other membrane components. In addition, it has so far not been excluded that the kinetics of O-antigen breakdown matches the timing of the phage particle opening step, the latter being highly temperature-sensitive [58]. As a consequence, Sf6 particles may rapidly clear off the *S. flexneri* serotype 2a O-antigen receptor whereas the irreversible phage cell surface adsorption cannot be completed at the given temperature [32]. It has also been shown for *Y. enterocolitica*-specific phages that expression of Omp-phage receptors was highly regulated by temperature [59].

When analyzing the O-antigen compositions of *S. flexneri* isolates used in this study, we found highly heterogeneous non-stoichiometric O-acetylation patterns that might be due to lysogens and consequently interfere with phage infection. O-Acetylation is conferred by O-acetyl transferases. The gene *oacB* encodes for an O-acetyltransferase which has been described to acetylate O3,4 on Rha*p*III and O6 on Glc*p*NAcIV leading to non-stoichiometric modification of *S. flexneri* O-antigen serotypes 1a, 1b, 2a, 5a, and Y [36,37]. OacB has been found in about 60% of all Y and in more than 90% of all serotype 2a strains*.* Sf6TSP recognized all different O-acetylation patterns found on Y and 2a strains in this study. Accordingly, the extended O-antigen binding site of Sf6TSP covered the epitopes of at least three common *S. flexneri* serotyping antibodies, i.e., group 3,4, type II, and factor 9 [60,61,62]. However, a complex O-antigen O-acetylation pattern might also interfere with the Sf6TSP endorhamnosidase activity as observed in one 2a strain analyzed in this work. Here, Sf6TSP could not fully break down the polysaccharide but produced a mixture of oligosaccharides with lengths between two and seven RUs. However, an earlier study had shown that Sf6TSP was unable to cleave O-antigen from serotype 2a LPS preparations [35]. This may be interpreted as additional O-acetylation hampering Sf6TSP cleavage. By contrast, in a recently isolated set of 16 new *S. flexneri* phages, a majority was active on serotype Y as well as on 2a [18]. This illustrates that during a local shigellosis outbreak these two serotypes were simultaneously addressed by bispecific phages, although the study did not analyze whether these phages used an O-antigen specific adsorption mechanism or whether other receptor interactions prevailed.

In conclusion, we can state that Sf6TSP was able to bind to strains with various O-acetylation patterns, although we have not analyzed their composition to molecular detail. An NMR analysis was hampered by the limited amounts of polysaccharides obtained from these strains. For determining the full range of ligands specifically bound by Sf6TSP, synthetic oligosaccharides are therefore a promising alternative. Synthesis provides defined glycan sequences and excludes heterogeneity [44]. Especially for *S. flexneri* 2a, synthetic O-antigen oligosaccharides are available from anti-*Shigella* vaccine design studies [61,63,64,65]. Our present work shows that in principle elongated synthetic fragments can be used for rapid quantification of Sf6TSP binding affinities. In future experiments, this system could be expanded to all types of O-acetylation patterns to precisely define the Sf6TSP specificity range for all variants. Furthermore, measurements on surfaces, with conjugates or in lipid bilayers are simplified using synthetic oligosaccharides [66,67].

### 4.2. Sf6TSP as a Pathogen Sensor for S. flexneri

Typically, *Shigella* spp. are identified by phenotyping on selective media and with slide agglutination serotyping tests. Moreover, in case of *S. flexneri,* genotyping targets the pINV B virulence plasmid, i.e., *ipg*D and *mxi*A as parts of the secretion and invasion machinery [68,69]. Combining these approaches usually can also distinguish the pathovars EIEC and *Shigella* spp. [70]. In the present work, we have established a microtiter plate-based ELITA as a useful tool which can be added to diagnostic laboratory procedures.

Usually, freshly grown *S. flexneri* adsorbed to the plate elicited high signals that did not vary between the different serotypes. This is in contrast with an ELITA test described earlier, using StrepII-P22TSP to screen a *Salmonella* library [24]. Here, different signal intensities were observed for different *Salmonella* Typhimurium strains. However, we did not quantify the number of bacterial cells that were adsorbed to the surface nor did we relate their adsorption behavior to the presence of proteinaceous adhesive components in the cell wall. Therefore, we cannot exclude that amplitude differences in the test are due to different number of immobilized bacteria. However, Strep-tagged TSP have been tested before as probes for detecting bacteria free in solution with flow cytometry [24]. Here, signal intensity variations found for different strains were similar to those observed in ELITA on the microtiter plate. From this data it was concluded that primarily the affinity of TSP towards the respective O-antigen dominates the intensity of the signal rather than the number of immobilized bacteria. Hence, as we only investigated four different *S. flexneri* strains, further test series should apply more extended strain libraries and quantify the numbers of surface-adsorbed bacteria. Diagnosis here may focus on serotype 2a strains, because serotype Y is not essential in the field. As a perspective, epidemiological surveillance could then match areas for a 2a serotype-specific vaccine.

Still, the tests outlined above require cultivation of bacteria and more rapid and direct detection tools are desirable to overcome this drawback. The Sf6TSP-NBD fluorescent sensor has the advantage that its signal amplitude increases upon binding of the *S. flexneri* O-antigen. This is in contrast to constitutively fluorescent probes that might in principle result in false positive results upon unspecific binding. Our proof-of-principle experiment with Sf6TSP-NBD and purified *S. flexneri* O-polysaccharide preparations was the starting point for optimizing the set-up in various directions. First, we note that, due to material limitations, we have not tested Sf6TSP-NBD on *S. flexneri* serotype 2a O-polysaccharide. In principle, Sf6TSP-NBD is not intended to distinguish serotypes Y and 2a, but should elicit a positive signal on both. Further developments thus should aim at isolating an exclusively 2a-specific bacteriophage TSP for that purpose [18].

Moreover, in the given test format, the different fluorescent signal-to-noise ratios at various protein concentrations have to be investigated. At the concentrations of Sf6TSP-NBD applied in our cuvette-based experiment, the half maximal concentration of *S. flexneri* serotype Y polysaccharide that could be detected was about 4 μg mL^−1^, which can be seen as the detection limit under the given conditions (*cf.* Figure 4b). With approximately 3.4% of the bacterial dry mass being LPS, a rough estimate yields about 4 fg polysaccharide per single bacterial cell [71,72]. Given a mean O-antigen chain length of 15 RUs in our preparation, this means that the amount detected with our set-up would correspond to roughly 1000 cfu mL^−1^ *S. flexneri*. This detection limit would not be sufficient to detect in the necessary count range of 10 cfu mL^−1^ as lowest infectious level for *S. flexneri* [11]. Further improvements could be made by increasing the labeling efficiency or by protein high affinity engineering [73]. Moreover, TSP could also be useful in concurrent enrichment on solid matrices, flow cytometry-based approaches or modified for immune-PCR [24,74].

## 5. Conclusions

Sf6TSP has proven to be applicable as an *S. flexneri* sensor both in microtiter plate-based ELITA and as a fluorescently engineered probe with high O-antigen specificity. We found that Sf6TSP bound to *S. flexneri* serotypes Y and 2a in various O-acetylation states. Our study shows that bacteriophages cell surface recognition proteins can be readily incorporated in different detection set-ups. With the growing number of phages isolated from various bacterial habitats this emphasizes the general importance also to characterize and collect their receptor binding proteins. With this, more specific and faster bacterial diagnostics tools can be obtained employing low technology assays as ELITA. Based on the high specificities found in bacteriophage receptor binding proteins, the resulting “tailtyping” procedures may be important in the future to combine with sero-, geno-, and lysotyping.

## Figures and Tables

**Table 1:** *Shigella flexneri* natural isolates characterized by slide agglutination with monospecific antibodies and PCR-based genetic analysis.

Bacterial Isolate ^1^	Anti-*S. flexneri* Monoclonal Antibody		PCR (*wzx*) ^2^
	Group 3,4 (Y)	Type 2	
*S. flexneri* Y 99-2001	+	−	+
*S. flexneri* Y 03-650	+	−	+
*S. flexneri* 2a 03-6557	+	+	+
*S. flexneri* 2a 08-7230	+	+	-

^1^ Nomenclature of bacterial strains is according to the numbering system of the Wernigerode collection; ^2^ Genotyping with primer sequences for the O-antigen polymerase gene *wzx* as published in Reference [49].

**Table 2:** *N*-methyl-*N*-[2-[methyl(7-nitro-2,1,3-benzoxadiazol-4-yl)amino]ethyl] (NBD)-fluorescent labelling of Sf6TSP E366A/D399A via cysteine residues.

Sf6 TSP E366A/D399A Cysteine Mutant	Labeling Efficiency %	NBD Fluorescence Amplitude Increase at 540 nm ^1^ %
V204C	19.4	−10
S246C	42.8	53
T315C	43.2	−15
N340C	66.7	78
Y400C	48.3	25
T443C	63.3	12

^1^ Signal change after addition of 24 μg mL^−1^ *S. flexneri* serotype Y polysaccharide to 184 nM NBD labeled Sf6TSP E366A/D399A cysteine mutant.

## Supplementary Materials

The following are available online at http://www.mdpi.com/1999-4915/10/8/431/s1, Figure S1: Chromatogram of digested SfY ScBp and MS data, Figure S2: Chromatogram of digested SfY 99–2001 polysaccharide and MS data, Figure S3: Chromatogram of digested SfY 03–650 polysaccharide and MS data, Figure S4: Chromatogram of digested Sf2a ScBp and MS data, Figure S5: Chromatogram of digested Sf2a 03–6557 polysaccharide and MS data, Figure S6: Chromatogram of Sf2a 08–7230 polysaccharide and MS data, Figure S7: Organic solvent stability of Sf6TSP, Figure S8: Binding kinetics of Sf6TSP N340C labeled with NBD to SfY polysaccharide, Table S1: Annotated masses from MALDI-TOF MS from digested SfY ScBp polysaccharide, Table S2: Annotated masses from MALDI-TOF MS from digested SfY 99–2001 polysaccharide, Table S3: Annotated masses from MALDI-TOF MS from digested SfY 03–650 polysaccharide, Table S4: Annotated masses from MALDI-TOF MS from digested Sf2a ScBp polysaccharide, Table S5: Annotated masses from MALDI-TOF MS from digested Sf2a 03–6557 polysaccharide, Table S6: Annotated masses from MALDI-TOF MS from digested Sf2a 08–7230 polysaccharide.
