# Supplementary material for: Bacteriophage Sf6 Tailspike Protein for Detection of Shigella flexneri Pathogens
Source: Viruses. 2018 Aug 15;10(8):431. doi: 10.3390/v10080431 (PMC6116271; doi:10.3390/v10080431)
Supplement: Supplementary file 1 [file viruses-10-00431-s001.zip › Kunstmann_SI_Viruses_Appl_Supplement.pdf]

viruses

Communication – Supplementary material

Bacteriophage Sf6 tailspike protein for detection of *Shigella flexneri* pathogens

Sonja Kunstmann [1], Tom Scheidt [1,#], Saskia Buchwald [1], Alexandra Helm [1], Laurence A. Mulard [2,3], Angelika Fruth [4], and Stefanie Barbirz [1,*]

[1] Physical Biochemistry, University of Potsdam, 14476 Potsdam, Germany; sonja.kunstmann@mpikg.mpg.de (S.K.); ts599@cam.ac.uk (T.S.); sb165329@uni-greifwald.de (S.Bu.); ahelm@uni-potsdam.de (A.H.)
[2] Institut Pasteur, Unité de Chimie des Biomolécules, 28 rue du Dr. Roux, 75724, Paris Cedex 15, France.
[3] CNRS UMR 3523, Institut Pasteur, 75015, Paris, France; laurence.mulard@pasteur.fr (L.M.)
[4] National Reference Centre for Salmonella and other Bacterial Enterics, Robert Koch Institute, 38855 Wernigerode, Germany; frutha@rki.de (A.F.)
* Correspondence: barbirz@uni-potsdam.de (S.B.); Tel.: +49-331-977-5322
\# Present address: Department of Chemistry, University of Cambridge, Cambridge,CB2 1EW, U.K.

Received: 29 May 2018; Accepted: date; Published: date

Supplementary Material – Content

Figure S1. Chromatogram of digested SfY ScBp and MS data 2
Figure S2. Chromatogram of digested SfY 99-2001 polysaccharide and MS data 3
Figure S3. Chromatogram of digested *S. flexneri* polysaccharide Y 03-650 4
Figure S4. Chromatogram of digested Sf2a ScBp and MS data 5
Figure S5. Chromatogram of digested Sf2a 03-6557 polysaccharide and MS data 9
Figure S6. Chromatogram of Sf2a 08-7230 polysaccharide and MS data 13
Figure S7. Organic solvent stability of Sf6TSP 17
Figure S8. Binding kinetics of Sf6TSP N340C labeled with NBD to SfY polysaccharide 17

Table S1. Annotated masses from MALDI-TOF MS from digested SfY ScBp polysaccharide 2
Table S2. Annotated masses from MALDI-TOF MS from digested SfY 99-2001 polysaccharide 3
Table S3. Annotated masses from MALDI-TOF MS from digested SfY 03-650 polysaccharide 4
Table S4. Annotated masses from MALDI-TOF MS from digested Sf2a ScBp polysaccharide...............................6
Table S5. Annotated masses from MALDI-TOF MS from digested Sf2a 03-6557 polysaccharide 10
Table S6. Annotated masses from MALDI-TOF MS from digested Sf2a 08-7230 polysaccharide 14

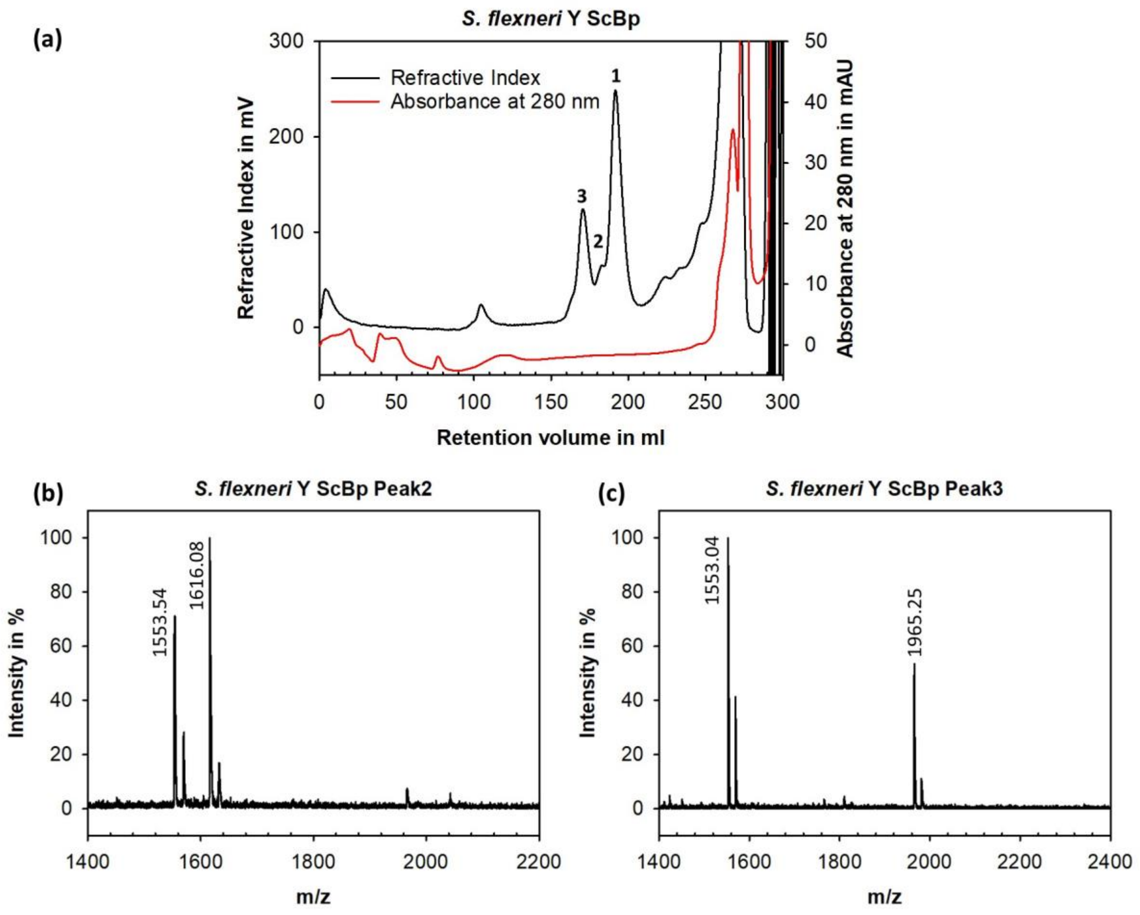

Figure S1. Chromatogram of digested SfY ScBp and MS data**. (a)** Chromatogram of digested *S. flexneri* polysaccharide Y ScBp with refractive index (black line) and absorption at 280 nm (red line). Numbers indicate the isolated peaks. **(b-c)** Mass spectrograms of the peak 2 and 3 from *S. flexneri* Y ScBp. Masses for non-acetylated peaks as sodium salt are indicated. Further peaks are listed in Table S1.

Table S1. Annotated masses from MALDI-TOF MS from digested SfY ScBp polysaccharide

Oligosaccharide (Oligosac)	Molecular formula	Calculated mass	Annotated mass	Intensity %Peak1	Intensity %Peak2	Intensity %Peak3
	C52O35H88N2	1300.52	-	-	-	-
**Octasac (8)**	$+Na^{+}$	1323.51	1323.79	100		
	$+K^{+}$	1339.48	-	-		
**Unidentified**			1553.05	-	66.0	100
			1569.14		26.4	40.6
**Decasac (10) (+2Rha)**	C64O43H108N2	1593.64	-	-	-	-
	$+Na^{+}$	1615.62	1616.08		100	
	$+K^{+}$	1631.60	1631.99		15.0	
	C78O52H131N3	1943.87	-	-	-	-
**Dodecasac (12)**	$+Na^{+}$	1965.86	1965.25			50.2
	$+K^{+}$	1981.96	1981.22			10.8

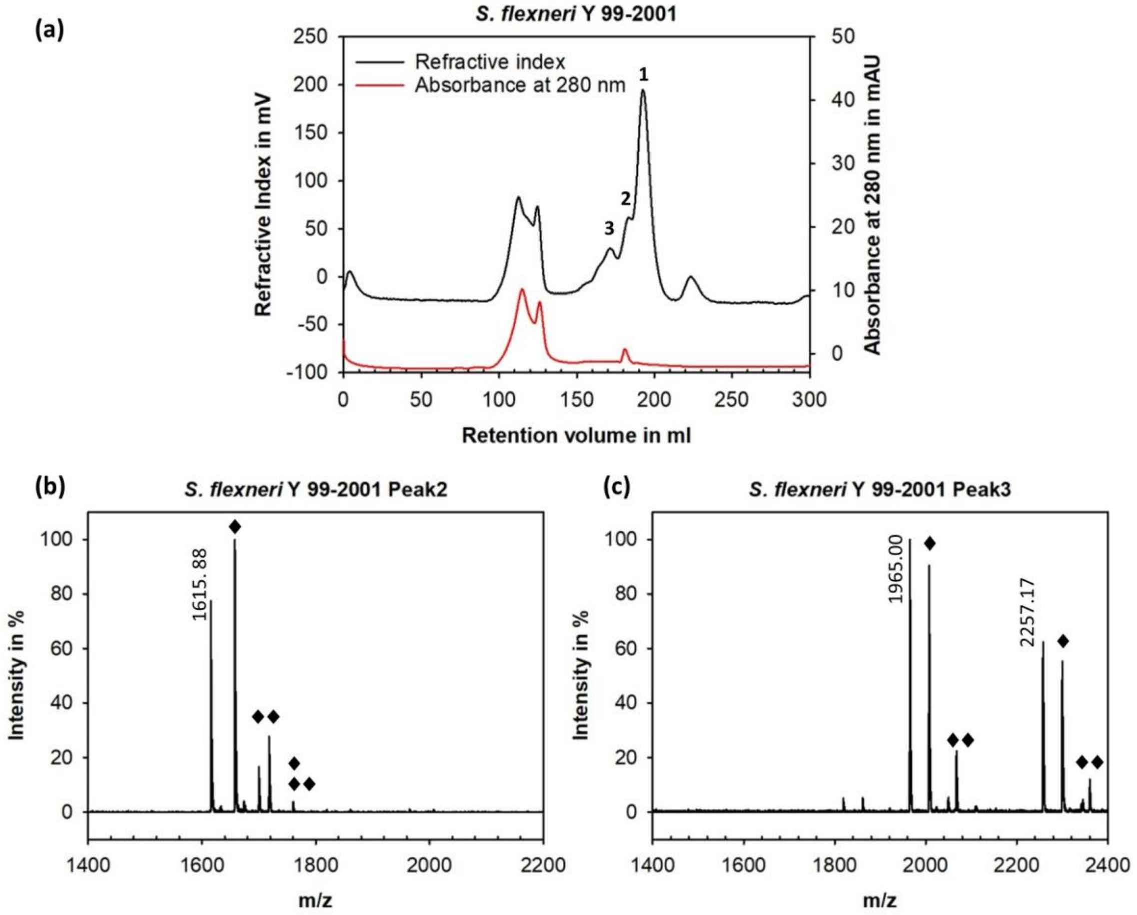

Figure S2. Chromatogram of digested SfY 99-2001 polysaccharide and MS data **(a)** Chromatogram of digested *S. flexneri* polysaccharide Y 99-2001 with refractive index (black line) and absorption at 280 nm (red line). Numbers indicate the isolated peaks. **(b-c)** Mass spectrograms of the peak 2 and 3 from *S. flexneri* Y 99-2001. Masses for non-acetylated peaks as sodium salt are indicated. Further peaks are listed in Table S2. Number of diamonds indicates the number of acetylations.

Table S2. Annotated masses from MALDI-TOF MS from digested SfY 99-2001 polysaccharide

Oligosaccharide (Oligosac)	Molecular formula	Calculated mass	Annotated mass	Intensity %Peak1	Intensity %Peak2	Intensity %Peak3
	C52O35H88N2	1300.52	-	-	-	-
**Octasac (8)**	$+Na^{+}$	1323.51	1323.70	100		
	$+K^{+}$	1339.48	1339.68	14.5		
	C54O36H90N2	1343.53	-	-	-	-
**Octasac -1Ac**	$+Na^{+}$	1365.52	1365.70	14.4		
	$+K^{+}$	1381.49	-	-		
	C64O43H108N2	1593.64	-	-	-	-
**Decasac (10) (+2Rha)**	$+Na^{+}$	1615.62	1615.83	8.41	77.8	
	$+K^{+}$	1631.60	-	-	-	
	C66O44H110N2	1635.65	-	-	-	-
**Decasac -1Ac**	$+Na^{+}$	1657.63	1657.93		100	
	$+K^{+}$	1673.61	-		-	
	C68O45H112N2	1678.61	-	-	-	-
**Decasac -2Ac**	$+Na^{+}$	1700.60	1699.90		16.4	
	$+K^{+}$	1716.70	1717.93		27.5	
**Dodecasac (12)**	C78O52H131N3	1943.87	-	-	-	-

	+Na$^+$	1965.86	1965.00			100
	+K$^+$	1981.96	-			-
**Dodecasac -1Ac**	C80O53H133N3	1985.91	-	-	-	-
	+Na$^+$	2007.89	2007.07			91.44
	+K$^+$	2024.00	-			-
**Dodecasac -2Ac**	C82O54H135N3	2027.95	-	-	-	-
	+Na$^+$	2049.93	-			-
	+K$^+$	2066.04	2067.08			20.9
**Tetrakaidecasac (14) (+2 Rha)**	C90O60H151N3	2236.16	-	-	-	-
	+Na$^+$	2258.14	2257.17			56.9
	+K$^+$	2274.25	-			-
**Tetrakaidecasac -1Ac**	C92O61H153N3	2278.19	-	-	-	-
	+Na$^+$	2300.18	2299.18			48.0
	+K$^+$	2316.28	-			-
**Tetrakaidecasac -2Ac**	C94O62H155N3	2320.23	-	-	-	-
	+Na$^+$	2342.21	-			-
	+K$^+$	2358.32	2359.20			9.17

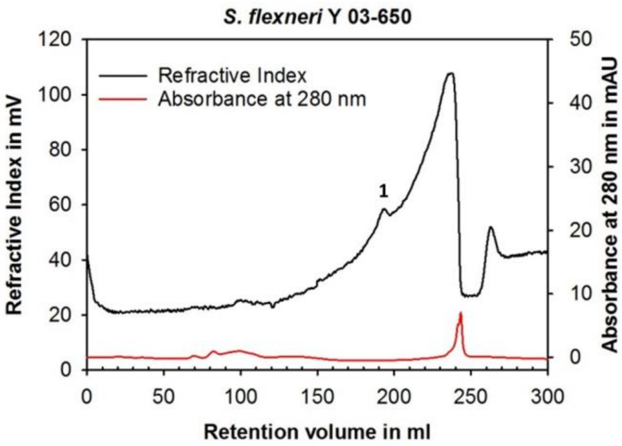

Figure S3. Chromatogram of digested *S. flexneri* polysaccharide Y 03-650. Refractive index (black line) and absorption at 280 nm (red line). Number indicates the isolated peak.

Table S3. Annotated masses from MALDI-TOF MS from digested SfY 03-650 polysaccharide

Oligosaccharide (Oligosac)	Molecular formula	Calculated mass	Annotated mass	Intensity% Peak1
**Heptasac (7) (2RU -1Rha)**	C46O31H78N2	1156.12	-	-
	+Na$^+$	1178.10	1177.69	8.50
	+K$^+$	1194.21	-	-
**Heptasac -1Ac**	C48O32H80N2	1198.15	-	-
	+Na$^+$	1220.13	1219.51	10.3
	+K$^+$	1236.24	-	-
**Octasac (8) (2RU)**	C52O35H88N2	1300.52	-	-
	+Na$^+$	1323.51	1323.60	100
	+K$^+$	1339.48	1339.96	28.6
**Octasac -1Ac**	C54O36H90N2	1343.53	-	-
	+Na$^+$	1365.52	1365.96	60.5
	+K$^+$	1381.49	1382.26	21.2
**Octasac -2Ac**	C56O37H92N2	1386.33	-	-
	+Na$^+$	1408.31	1408.09	32.4
	+K$^+$	1424.42	1424.96	6.62

**Nonasac (2RU +1Rha)**	C58O39H98N2	1448.40	-	-
	$+Na^+$	1470.38	1469.98	5.29
	$+K^+$	1486.49	-	-
**Decasac (10) (2RU +2Rha)**	C64O43H108N2	1593.64	-	-
	$+Na^+$	1615.62	1616.36	27.2
	$+K^+$	1631.60	1632.31	5.89
**Decasac -1Ac**	C66O44H110N2	1635.65	-	-
	$+Na^+$	1657.63	1658.41	11.0
	$+K^+$	1673.61	-	-
**Undefined**			1691.47	26.4

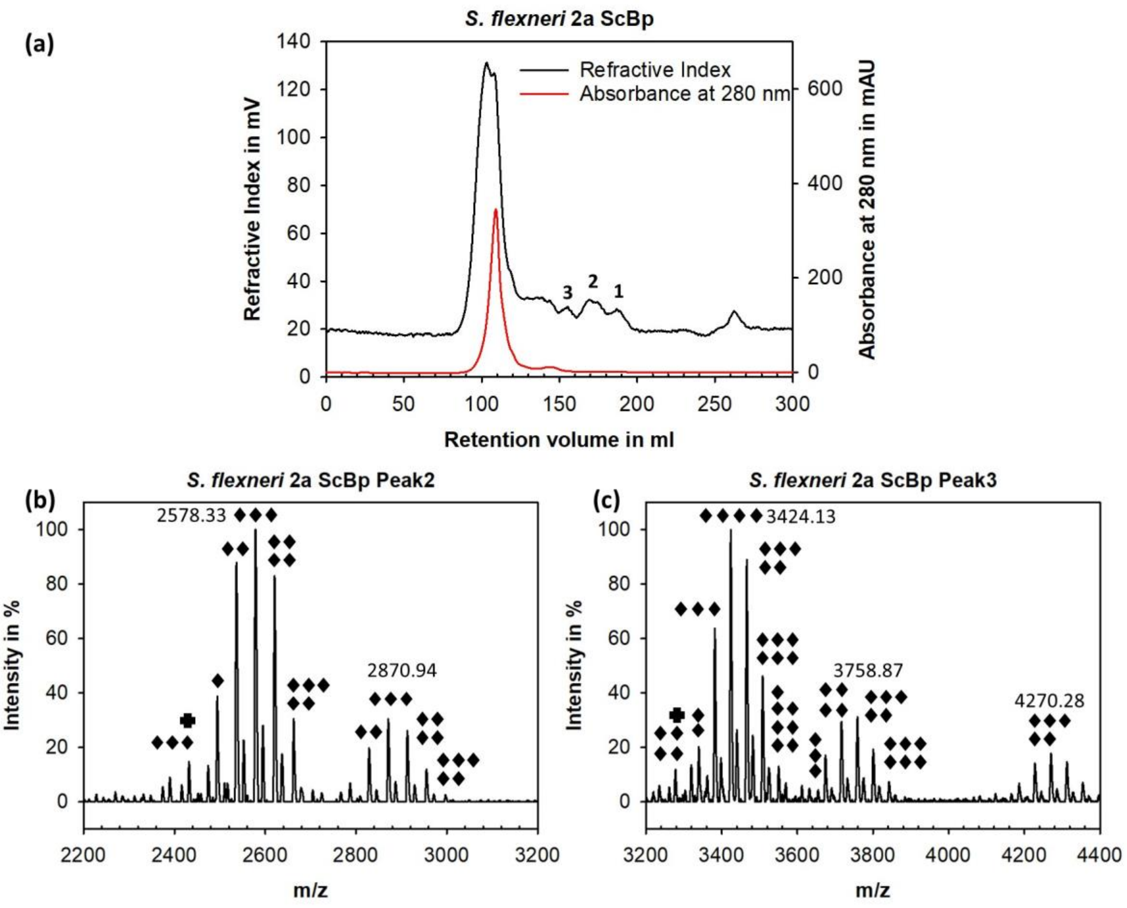

Figure S4. Chromatogram of digested Sf2a ScBp and MS data. **(a)** Chromatogram of digested *S. flexneri* polysaccharide 2a ScBp with refractive index (black line) and absorption at 280 nm (red line). Numbers indicate the isolated peaks. **(b-c)** Mass spectrograms of the peak 2 and 3 from *S. flexneri* 2a ScBp. Masses for the most populated acetylated peak as sodium salt are indicated. Further peaks are listed in Table S 4. Number of diamonds indicates the number of acetylation. Plus = Deglucosylated.

Table S4. Annotated masses from MALDI-TOF MS from digested Sf2a ScBp polysaccharide

Oligosaccharide (Oligosac)	Molecular formula	Mass (calc.)	Mass (exp.)	Intensity% Peak1	Intensity% Peak2	Intensity% Peak3
**Decasac (10)**	C64O45H108N2	1626.54	-	-	-	-
**(2RU)**	+Na$^+$	1648.52	1648.03	34.8		
	+K$^+$	1664.63	1664.17	7.17		
**Decasac**	C66O46H110N2	1668.58	-	-	-	-
**-1Ac**	+Na$^+$	1690.57	1689.68	92.6		
	+K$^+$	1706.67	1706.14	23.0		
**Decasac**	C68O47H112N2	1710.61	-	-	-	
**-2Ac**	+Na$^+$	1732.59	1731.69	100		
	+K$^+$	1748.70	1748.40	25.3		
**Decasac**	C70O48H114N2	1752.65	-	-	-	
**-3Ac**	+Na$^+$	1774.63	1774.30	53.8	5.12	
	+K$^+$	1790.74	1790.14	11.2	-	
**Decasac**	C72O49H116N2	1794.69	-	-	-	
**-4Ac**	+Na$^+$	1816.67	1816.63	10.3		
	+K$^+$	1832.78	-	-		
**Dodecasac (12)**	C78O54H130N2	1960.86	-	-	-	
**-1Ac**	+Na$^+$	1982.84	1982.52	20.9		
**(2RU +2Rha)**	+K$^+$	1998.95	1998.51	5.17		
**Dodecasac**	C80O55H132N2	2002.90	-	-	-	
**-2Ac**	+Na$^+$	2024.88	2024.52	31.0	13.4	
	+K$^+$	2040.99	2040.42	7.99	-	
**Dodecasac**	C82O56H134N2	2044.93	-	-	-	
**-3Ac**	+Na$^+$	2066.91	2066.62	18.4	21.1	
	+K$^+$	2083.02	-	-	6.09	
**Dodecasac**	C84O57H136N2	2086.97	-	-	-	
**-4Ac**	+Na$^+$	2108.95	2108.72		12.52	
	+K$^+$	2125.06	-		-	
**Tetrakaidecasac**	C94O64H155N3	2352.23	-	-	-	-
**(14)-2Ac**	+Na$^+$	2374.21	2374.43		5.55	
**(3RU -1Glc)**	+K$^+$	2390.32	2390.15		9.01	
**Tetrakaidecasac**	C96O65H157N3	2394.27	-		-	
**-3Ac**	+Na$^+$	2416.25	2416.14		6.24	
	+K$^+$	2432.36	2431.86		14.8	
**Tetrakaidecasac**	C98O66H159N3	2436.30	-	-	-	
**-4Ac**	+Na$^+$	2458.28	-		-	
	+K$^+$	2474.39	2474.30		13.3	
**Tetrakaidecasac**	C100O67H161N3	2478.34	-	-	-	
**-5Ac**	+Na$^+$	2500.32	-		-	
	+K$^+$	2516.43	2516.83		6.78	
**Pentakaidecasac**	C98O68H163N3	2472.33	-	-	-	-
**(15)-1Ac**	+Na$^+$	2494.32	2494.61		38.7	
**(3RU)**	+K$^+$	2510.42	2510.48		6.84	
**Pentakaidecasac**	C100O69H165N3	2514.37	-	-	-	-
**-2Ac**	+Na$^+$	2536.35	2536.41		87.9	
	+K$^+$	2552.46	2552.28		22.6	
**Pentakaidecasac**	C102O70H167N3	2556.41	-	-	-	-
**-3Ac**	+Na$^+$	2578.39	2578.33		100	
	+K$^+$	2594.50	2594.47		28.0	
**Pentakaidecasac**	C104O71H169N3	2598.44	-	-	-	-

**-4Ac**	+Na$^+$	2620.43	2620.45		83.0	
	+K$^+$	2636.53	2636.72		17.6	
**Pentakaidecasac -5Ac**	C106O72H171N3	2640.48	-	-	-	
	+Na$^+$	2662.46	2662.62		30.4	
	+K$^+$	2678.57	2678.68		5.20	
**Heptakaidecasac**	C110O76H183N3	2764.62	-	-	-	
**(17)-1Ac**	+Na$^+$	2786.60	2786.65		6.79	
**(3RU +2Rha)**	+K$^+$	2802.71	-		-	
**Heptakaidecasac -2Ac**	C112O77H185N3	2806.65	-	-	-	
	+Na$^+$	2828.64	2829.04		19.76	
	+K$^+$	2844.74			-	
**Heptakaidecasac -3Ac**	C114O78H187N3	2848.69	-	-	-	
	+Na$^+$	2870.67	2870.94		30.5	
	+K$^+$	2886.78	2887.27		7.43	
**Heptakaidecasac -4Ac**	C116O79H189N3	2890.73	-	-	-	
	+Na$^+$	2912.71	2913.11		26.1	
	+K$^+$	2928.82	2928.99		6.12	
**Heptakaidecasac -5Ac**	C118O80H191N3	2932.76	-	-	-	-
	+Na$^+$	2954.75	2955.17		11.9	5.06
	+K$^+$	2970.85	-		-	-
**Nonakaidecasac**	C130O88H212N4	3240.06	-	-	-	-
**(19)-4Ac**	+Na$^+$	3262.04	-			-
**(4RU -1Glc)**	+K$^+$	3278.15	3278.14			11.6
**Nonakaidecasac -5Ac**	C132O89H214N4	3282.10	-	-	-	-
	+Na$^+$	3304.08	-			-
	+K$^+$	3320.19	3319.52			13.4
**Nonakaidecasac -6Ac**	C134O90H216N4	3324.13	-	-	-	-
	+Na$^+$	3346.12	-			-
	+K$^+$	3362.23	3362.46			9.44
**Icosasac**	C132O91H218N4	3318.13	-	-	-	-
**(20)-2Ac**	+Na$^+$	3340.11	3340.01			20.0
**(4RU)**	+K$^+$	3356.22	-			-
**Icosasac -3Ac**	C134O92H220N4	3360.16	-	-	-	-
	+Na$^+$	3382.15	3381.99		9.10	63.7
	+K$^+$	3398.26	3398.18		-	15.9
**Icosasac -4Ac**	C136O93H222N4	3402.20	-	-	-	-
	+Na$^+$	3424.18	3424.13		9.92	100
	+K$^+$	3440.29	3440.37		-	26.3
**Icosasac -5Ac**	C138O94H224N4	3444.24	-	-	-	-
	+Na$^+$	3466.22	3466.42		6.16	89.0
	+K$^+$	3482.33	3482.32		-	24.2
**Icosasac -6Ac**	C140O95H226N4	3486.28	-	-	-	-
	+Na$^+$	3508.26	3508.20			46.1
	+K$^+$	3524.37	3524.63			12.3
**Icosasac -7Ac**	C142O96H228N4	3528.31		-	-	-
	+Na$^+$	3550.29	3551.06			12.8
	+K$^+$	3566.40	-			-
**Doicosasac**	C146O100H240N4	3652.45	-	-	-	-
**(22)-3Ac**	+Na$^+$	3674.43	3674.44			16.9
**(4RU +2Rha)**	+K$^+$	3690.54	-			-
**Doicosasac**	C148O101H242N4	3694.48	-	-	-	-
**-4Ac**	+Na$^+$	3716.47	3717.17			29.2

	+K+	3732.58	3732.42			8.44
**Doicosasac -5Ac**	C150O102H244N4	3736.52	-	-	-	-
	+Na+	3758.50	3758.87			31.1
	+K+	3774.61	3775.25			8.62
**Doicosasac -6Ac**	C152O103H246N4	3778.56	-	-	-	-
	+Na+	3800.54	3800.58			19.2
	+K+	3816.65	3816.12			5.65
**Doicosasac -7Ac**	C154O104H248N4	3820.60	-	-	-	-
	+Na+	3842.58	3842.00			7.19
	+K+	3858.69	-			-
**Pentaicosasac (25)-3Ac (5RU)**	C166O114H272N5	4162.92	-	-	-	-
	+Na+	4184.90	4186.37			7.19
	+K+	4201.01	-			-
**Pentaicosasac -4Ac**	C168O115H274N5	4204.95	-	-	-	-
	+Na+	4226.93	4228.07			14.0
	+K+	4243.04	-			-
**Pentaicosasac -5Ac**	C170O116H276N5	4246.99	-	-	-	-
	+Na+	4268.97	4270.28			17.8
	+K+	4285.08	-			-
**Pentaicosasac -6Ac**	C172O117H278N5	4289.03	-	-	-	-
	+Na+	4311.01	4312.89			14.6
	+K+	4327.12	-			-
**Pentaicosasac -7Ac**	C174O118H280N5	4331.06	-	-	-	-
	+Na+	4353.04	4354.66			7.00
	+K+	4369.15	-			-

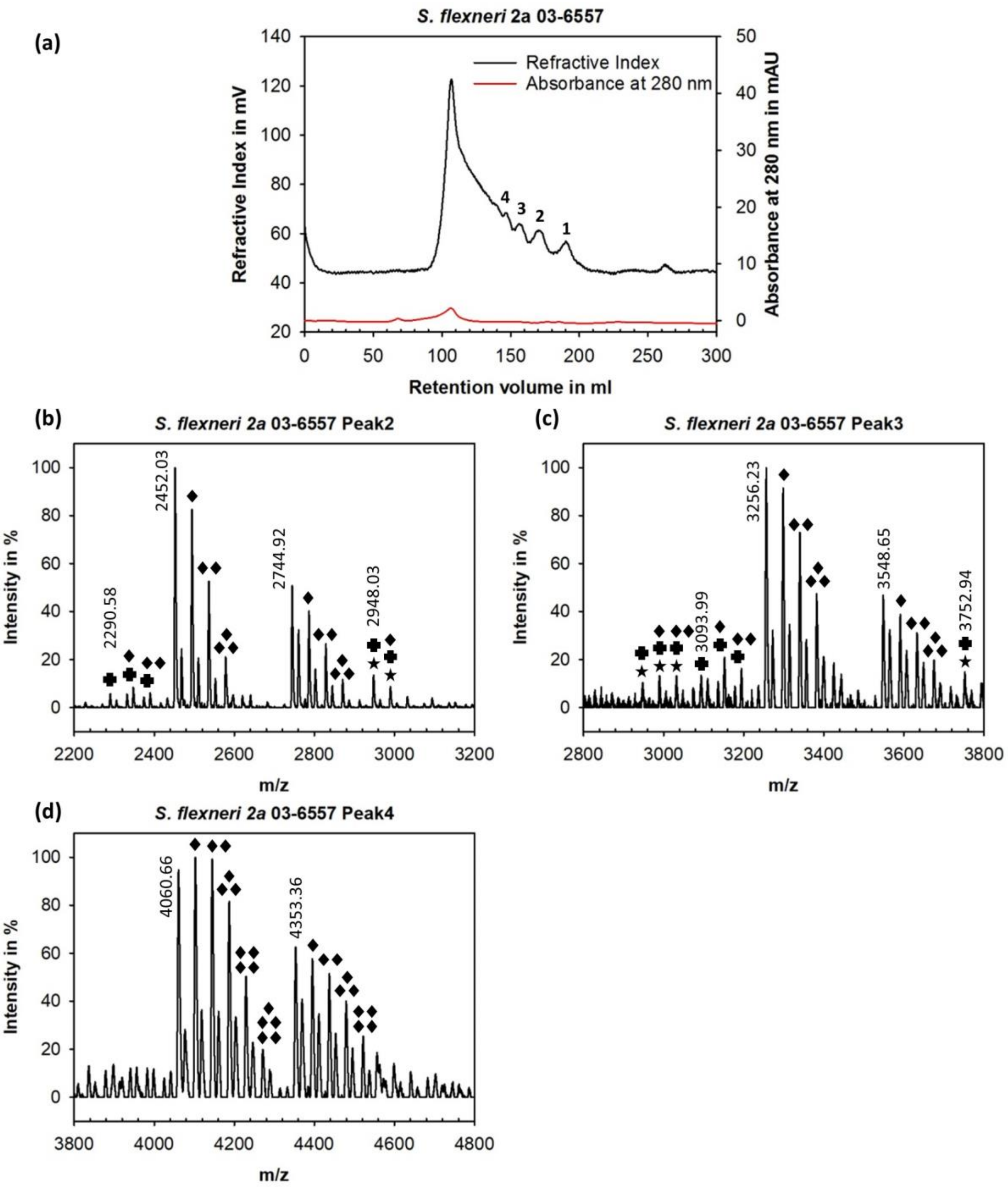

Figure S5. Chromatogram of digested Sf2a 03-6557 polysaccharide and MS data **(a)** Chromatogram with refractive index (black line) and absorption at 280 nm (red line). Numbers indicate the isolated peaks. **(b-d)** Mass spectrograms of *S. flexneri* 2a 03-6557. Masses for non-acetylated peaks as sodium salt are indicated. Further peaks are listed in Table S5. Diamond=Acetylation. Plus=Loss of Glc. Star=Loss of Rha.

Table S5. Annotated masses from MALDI-TOF MS from digested Sf2a 03-6557 polysaccharide

Oligosaccharide (Oligosac)	Molecular formula	Mass (calc.)	Mass (exp.)	Int.% Peak1	Int.% Peak2	Int.% Peak3	Int.% Peak4
**Nonasac (9) (2RU- 1Glc)**	C58O40H98N2	1464.40	-	-	-	-	-
	+Na+	1486.38	1486.29	11.9			
	+K+	1502.49	-	-			
**Nonasac -1Ac**	C60O41H100N2	1506.43	-	-	-	-	-
	+Na+	1528.42	1528.30	10.4			
	+K+	1544.53	1543.96	12.1			
**Nonasac -2Ac**	C62O42H102N2	1548.47	-	-	-	-	-
	+Na+	1570.45	1570.16	5.47			
	+K+	1586.56	1586.18	6.56			
**Decasac (10) (2RU)**	C64O45H108N2	1626.54	-	-	-	-	-
	+Na+	1648.52	1647.91	100			
	+K+	1664.63	1664.17	23.43			
**Decasac -1Ac**	C66O46H110N2	1668.58	-	-	-	-	-
	+Na+	1690.57	1690.37	71.5			
	+K+	1706.67	1706.22	14.2			
**Decasac -2Ac**	C68O47H112N2	1710.61	-	-	-	-	-
	+Na+	1732.59	1732.51	32.8			
	+K+	1748.70	-	-			
**Decasac -3Ac**	C70O48H114N2	1752.65	-	-	-	-	-
	+Na+	1774.63	1774.62	7.20			
	+K+	1790.74	-	-			
**Dodecasac (12) (2RU +2Rha)**	C76O53H128N2	1918.82	-	-	-	-	-
	+Na+	1940.80	1940.64	38.59			
	+K+	1956.91	1956.58	22.78			
**Dodecasac -1Ac**	C78O54H130N2	1960.86	-	-	-	-	-
	+Na+	1982.84	1982.56	27.1			
	+K+	1998.95	1998.60	10.6			
**Dodecasac -2Ac**	C80O55H132N2	2002.90	-	-	-	-	-
	+Na+	2024.88	2024.73	13.8			
	+K+	2040.99	-	-			
**Trikaidecasac (13) (3RU -1Glc -Rha)**	C84O58H141N3	2122.01	-	-	-	-	-
	+Na+	2144.00	2144.07	9.64			
	+K+	2160.10	-	-			
**Tetrakaidecasac (14) (3RU –Glc)**	C90O62H151N3	2268.16	-	-	-	-	-
	+Na+	2290.14	2290.58		5.70		
	+K+	2306.25	-		-		
**Tetrakaidecasac -1Ac**	C92O63H153N3	2310.19	-	-	-	-	-
	+Na+	2332.17	2332.36		5.50		
	+K+	2348.28	2347.76		8.32		
**Tetrakaidecasac -2Ac**	C94O64H155N3	2352.23	-	-	-	-	-
	+Na+	2374.21	-		-		
	+K+	2390.32	2390.10		6.02		
**Pentakaidecasac (15) (3RU)**	C96O67H161N3	2430.30	-	-	-	-	-
	+Na+	2452.28	2452.03	14.66	100		
	+K+	2468.39	2468.56	-	24.4		
**Pentakaidecasac -1Ac**	C98O68H163N3	2472.33	-	-	-	-	-
	+Na+	2494.32	2494.18	6.99	82.6		
	+K+	2510.42	2510.71	-	20.5		
**Pentakaidecasac**	C100O69H165N3	2514.37	-	-	-	-	-

**-2Ac**	+Na$^+$	2536.35	2536.65		52.6		
	+K$^+$	2552.46	2552.70		12.0		
**Pentakaidecasac -3Ac**	C102O70H167N3	2556.41	-	-	-	-	-
	+Na$^+$	2578.39	2578.71		21.0		
	+K$^+$	2594.50	-		-		
**Heptakaidecasac (17) (3RU +2Rha)**	C108O75H181N3	2722.58	-	-	-	-	-
	+Na$^+$	2744.56	2744.92		50.9		
	+K$^+$	2760.67	2760.69		32.3		
**Heptakaidecasac -1Ac**	C110O76H183N3	2764.62	-	-	-	-	-
	+Na$^+$	2786.60	2786.70		40.1		
	+K$^+$	2802.71	2802.78		16.0		
**Heptakaidecasac -2Ac**	C112O77H185N3	2806.65		-	-	-	-
	+Na$^+$	2828.64	2829.04		26.5		
	+K$^+$	2844.74	2844.89		9.00		
**Heptakaidecasac -3Ac**	C114O78H187N3	2848.69	-	-	-	-	-
	+Na$^+$	2870.67	2871.19		11.7		
	+K$^+$	2886.78	-		-		
**Octakaidecasac (18) (4RU -1Glc -1Rha)**	C116O80H194N4	2925.77	-	-	-	-	-
	+Na$^+$	2947.75	2948.03		13.4		
	+K$^+$	2963.86	-		-		
**Octakaidecasac -1Ac**	C118O81H196N4	2967.81	-	-	-	-	-
	+Na$^+$	2989.79	2989.63		8.69	13.2	
	+K$^+$	3005.90	-		-	-	
**Octakaidecasac -2Ac**	C120O82H198N4	3009.85	-	-	-	-	-
	+Na$^+$	3031.83	3032.24			13.1	
	+K$^+$	3047.94	-			-	
**Nonakaidecasac (19) (4RU -1Glc)**	C122O84H204N4	3071.91	-	-	-	-	-
	+Na$^+$	3093.90	3093.99			13.4	
	+K$^+$	3110.00	3109.73			12.0	
**Nonakaidecasac -1Ac**	C124O85H206N4	3113.95	-	-	-	-	-
	+Na$^+$	3135.93	3136.45			10.9	
	+K$^+$	3152.04	3152.25			21.1	
**Nonakaidecasac -2Ac**	C126O86H208N4	3155.99	-	-	-	-	-
	+Na$^+$	3177.97	-			-	
	+K$^+$	3194.08	3194.47			16.2	
**Icosasac (20) (4RU)**	C128O89H214N4	3234.05	-	-	-	-	-
	+Na$^+$	3256.04	3256.23		27.8	100	
	+K$^+$	3273.14	3272.49		-	31.7	
**Icosasac -1Ac**	C130O90H216N4	3276.09	-	-	-	-	-
	+Na$^+$	3298.07	3298.88		16.1	91.3	
	+K$^+$	3314.18	3314.86		-	34.4	
**Icosasac -2Ac**	C132O91H218N4	3318.13	-	-	-	-	-
	+Na$^+$	3340.11	3340.39		8.72	73.1	
	+K$^+$	3356.22	3356.58		-	28.3	
**Icosasac -3Ac**	C134O92H220N4	3360.16	-	-	-	-	-
	+Na$^+$	3382.15	3382.65			47.59	
	+K$^+$	3398.26	-			-	
**Doicosasac (22) (4RU +2Rha)**	C140O97H234N4	3526.34	-	-	-	-	-
	+Na$^+$	3548.32	3548.65			46.8	
	+K$^+$	3564.43	3565.24			32.1	
**Doicosasac -1Ac**	C142O98H236N4	3568.37	-	-	-	-	-
	+Na$^+$	3590.36	3591.32			38.5	

	+K$^+$	3606.46	3607.32			23.5	
**Doicosasac -2Ac**	C144O99H238N4	3610.41	-	-	-	-	-
	+Na$^+$	3632.39	3632.87			31.0	
	+K$^+$	3648.50	3649.43			18.7	
**Doicosasac -3Ac**	C146O100H240N4	3652.45	-	-	-	-	-
	+Na$^+$	3674.43	3675.53			19.8	
	+K$^+$	3690.54	3690.64			10.3	
**Triicosasac (23) (5RU -1Glc -1Rha)**	C148O102H247N5	3729.53	-	-	-	-	-
	+Na$^+$	3751.51	3752.94			14.7	
	+K$^+$	3767.62				-	
**Triicosasac -1Ac**	C150O103H249N5	3771.57	-	-	-	-	-
	+Na$^+$	3793.55	3795.31			10.5	
	+K$^+$	3809.66	-			-	
**Pentaicosasac (25) (5RU)**	C160O111H267N5	4037.81	-	-	-	-	-
	+Na$^+$	4059.79	4060.66			38.2	97.2
	+K$^+$	4075.90	4077.26			14.3	24.5
**Pentaicosasac -1Ac**	C162O112H268N5	4078.84	-	-	-	-	-
	+Na$^+$	4100.82	4102.69			27.7	100
	+K$^+$	4116.93	4118.29			11.0	32.0
**Pentaicosasac -2Ac**	C164O113H270N5	4120.88	-	-	-	-	-
	+Na$^+$	4142.86	4144.81			19.0	99.8
	+K$^+$	4158.97	4160.87			-	32.2
**Pentaicosasac -3Ac**	C166O114H272N5	4162.92	-	-	-	-	-
	+Na$^+$	4184.90	4186.98			10.7	82.1
	+K$^+$	4201.01	4203.11			-	31.0
**Pentaicosasac -4Ac**	C168O115H274N5	4204.95	-	-	-	-	-
	+Na$^+$	4226.93	4229.35				50.2
	+K$^+$	4243.04	4246.30				21.4
**Pentaicosasac -5Ac**	C170O116H276N5	4246.99		-	-	-	-
	+Na$^+$	4268.97	4271.94				19.1
	+K$^+$	4285.08	-				-
**Heptaicosasac (27) (5RU +2Rha)**	C172O119H287N5	4330.10	-	-	-	-	-
	+Na$^+$	4352.08	4353.36			9.47	64.4
	+K$^+$	4368.19	4368.94			8.05	40.0
**Hepaticosasac -1Ac**	C174O120H289N5	4372.13	-	-	-	-	-
	+Na$^+$	4394.11	4395.51				56.7
	+K$^+$	4410.22	4411.36				32.1
**Heptaicosasac -2Ac**	C176O121H291N5	4414.17	-	-	-	-	-
	+Na$^+$	4436.15	4437.68				50.8
	+K$^+$	4452.26	4453.10				24.7
**Heptaicosasac -3Ac**	C178O122H293N5	4456.21	-	-	-	-	-
	+Na$^+$	4478.19	4479.61				40.08
	+K$^+$	4494.30	4495.54				19.76
**Heptaicosasac -4Ac**	C180O123H295N5	4498.24	-	-	-	-	-
	+Na$^+$	4520.22	4522.24				24.9
	+K$^+$	4536.33	4537.49				9.63
**Triakonsac (30) (6RU)**	C192O133H320N6	4841.57	-	-	-	-	-
	+Na$^+$	4863.55	4865.78				44.1
	+K$^+$	4879.66	4881.67				13.6
**Triakonsac -1Ac**	C194O134H322N6	4883.61	-	-	-	-	-
	+Na$^+$	4905.59	4907.45				32.0
	+K$^+$	4921.70	4932.87				9.78

**Triakonsac -2Ac**	C196O135H324N6	4925.64	-	-	-	-	-
	$+Na^+$	4947.63	4949.37				24.3
	$+K^+$	4963.73	4964.93				6.00
**Triakonsac -3Ac**	C198O136H326N6	4967.68	-	-	-	-	-
	$+Na^+$	4989.66	4992.33				16.5
	$+K^+$	5005.77	-				-

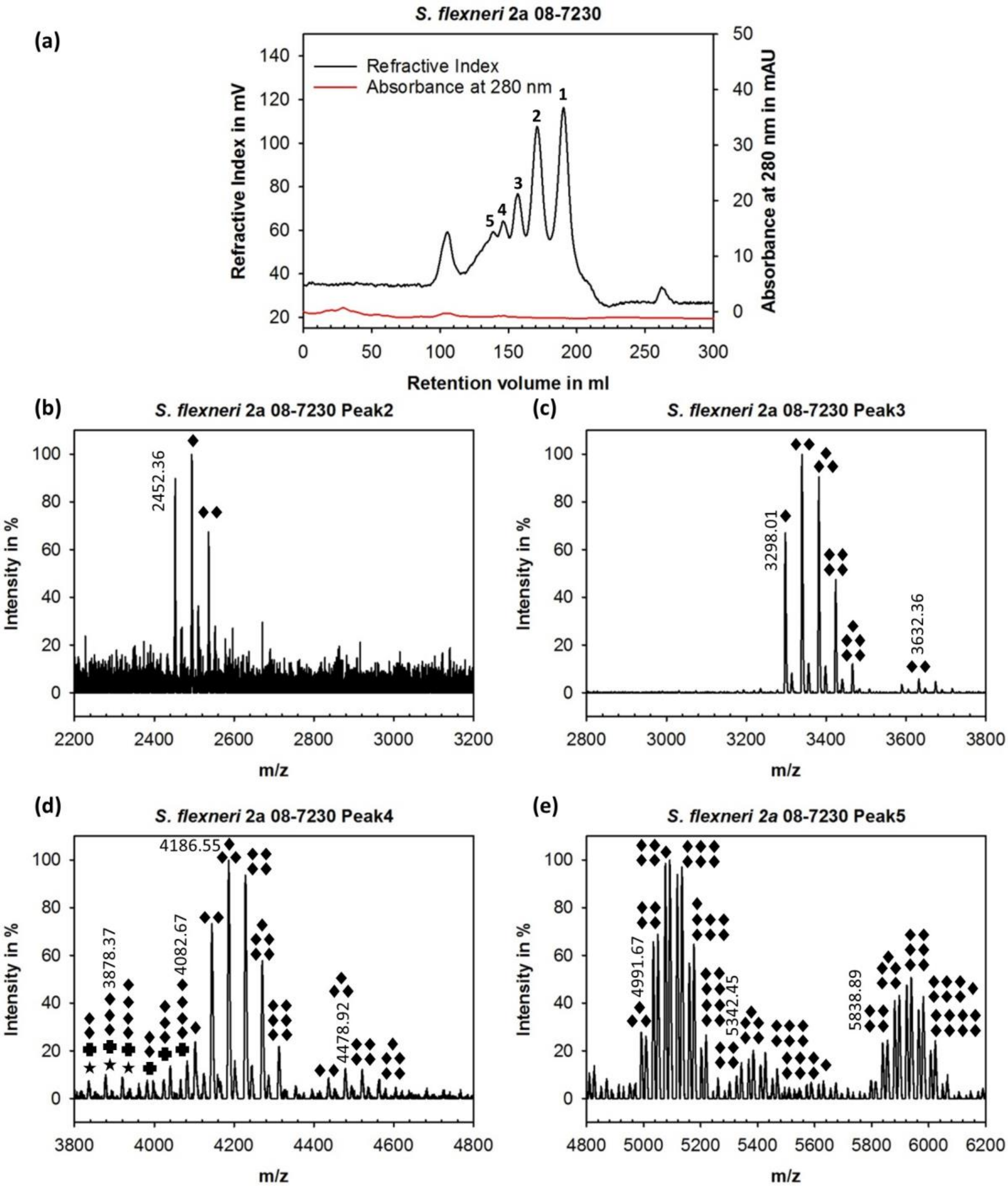

Figure S6. Chromatogram of Sf2a 08-7230 polysaccharide and MS data. **(a)** Chromatogram of digested *S. flexneri* polysaccharide 2a 08-7230 with refractive index (black line) and absorption at 280 nm (red line). Numbers indicate the isolated peaks. **(b-e)** Mass spectrograms of the peak 2-5 from *S. flexneri* 2a 08-7230. Masses for least acetylated oligosaccharide peaks as sodium salt are indicated. Further peaks are listed in Table S6. Number of diamonds indicates the number of acetylation. Plus = Loss of one glucose. Star = Loss of one rhamnose.

Table S6. Annotated masses from MALDI-TOF MS from digested Sf2a 08-7230 polysaccharide

Oligosac	Molecular formula	Mass (calc.)	Mass (exp.)	Int.% Peak 1	Int.% Peak 2	Int.% Peak 3	Int.% Peak 4	Int.% Peak 5
**Nonasac-1**	C60O41H100N2	1506.43	-	-	-	-	-	-
**Ac (9)**	+Na$^+$	1528.42	-	-				
**(2RU-1Glc)**	+K$^+$	1544.53	1544.47	10.0				
**Decasac**	C64O45H108N2	1626.54	-	-	-	-	-	-
**(10)**	+Na$^+$	1648.52	1647.61	100				
**(2RU)**	+K$^+$	1664.63	1664.44	8.68				
**Decasac -1Ac**	C66O46H110N2	1668.58	-	-	-	-	-	-
	+Na$^+$	1690.57	1689.72	51.2				
	+K$^+$	1706.67	1706.22	5.87				
**Decasac -2Ac**	C68O47H112N2	1710.61	-	-	-	-	-	-
	+Na$^+$	1732.59	1732.36	23.4				
	+K$^+$	1748.70	-	-				
**Decasac -3Ac**	C70O48H114N2	1752.65	-	-	-	-	-	-
	+Na$^+$	1774.63	1775.41	7.09				
	+K$^+$	1790.74	-	-				
**Pentakaide**	C96O67H161N3	2430.30	-	-	-	-	-	-
**casac (15)**	+Na$^+$	2452.28	2452.36		89.9			
**(3RU)**	+K$^+$	2468.39	2469.59		27.4			
**Pentakaide**	C98O68H163N3	2472.33	-	-	-	-	-	-
**casac**	+Na$^+$	2494.32	2493.62		100			
**-1Ac**	+K$^+$	2510.42	2510.62		36.4			
**Pentakaide**	C100O69H165N3	2514.37	-	-	-	-	-	-
**casac**	+Na$^+$	2536.35	2536.46		67.5			
**-2Ac**	+K$^+$	2552.46	2552.70		28.1			
**Icosasac**	C130O90H216N4	3276.09	-	-	-	-	-	-
**(20)-1Ac**	+Na$^+$	3298.07	3298.01			67.1		
**(4RU)**	+K$^+$	3314.18	3314.11			8.32		
**Icosasac -2Ac**	C132O91H218N4	3318.13	-	-	-	-	-	-
	+Na$^+$	3340.11	3340.17			100	11.6	
	+K$^+$	3356.22	3356.09			12.4	-	
**Icosasac -3Ac**	C134O92H220N4	3360.16	-	-	-	-	-	-
	+Na$^+$	3382.15	3382.16			90.5	14.1	
	+K$^+$	3398.26	3398.84			11.3	-	
**Icosasac -4Ac**	C136O93H222N4	3402.20	-	-	-	-	-	-
	+Na$^+$	3424.18	3424.19			47.6	14.6	
	+K$^+$	3440.29	3440.47			5.65	-	
**Icosasac -5Ac**	C138O94H224N4	3444.24	-	-	-	-	-	-
	+Na$^+$	3466.22	3466.42			12.3	9.52	
	+K$^+$	3482.33	-			-	-	
**Doicosasac**	C144O99H238N4	3610.41	-	-	-	-	-	-
**(22)-2Ac**	+Na$^+$	3632.39	3632.36			5.87	8.67	
**4RU+2Rha**	+K$^+$	3648.50	-			-	-	
**Doicosasac -3Ac**	C146O100H240N4	3652.45	-	-	-	-	-	-
	+Na$^+$	3674.43	3674.39				11.8	
	+K$^+$	3690.54	-				-	
**Doicosasac -4Ac**	C148O101H242N4	3694.48	-	-	-	-	-	-
	+Na$^+$	3716.47	3716.60				12.2	
	+K$^+$	3732.58	-				-	

**Triicosasac (23)-2Ac 5RU-1Glc-1Rha**	C152O104H251N5	3813.60	-	-	-	-	-	-
	$+Na^{+}$	3835.59	3836.35				7.48	
	$+K^{+}$	3851.69	-				-	
**Triicosasac -3Ac**	C154O105H253N5	3855.64	-	-	-	-	-	-
	$+Na^{+}$	3877.62	3878.37				10.1	
	$+K^{+}$	3893.73	-				-	
**Triicosasac -4Ac**	C156O106H255N5	3897.68	-	-	-	-	-	-
	$+Na^{+}$	3919.66	3919.62				9.00	
	$+K^{+}$	3935.77	-				-	
**Tetraicosasac (24)-2Ac (5RU-1Glc)**	C158O108H261N5	3959.75	-	-	-	-	-	-
	$+Na^{+}$	3981.73	3982.53				7.78	
	$+K^{+}$	3997.84	3996.95				7.64	
**Tetraicosasac-3Ac**	C160O109H263N5	4001.78	-	-	-	-	-	-
	$+Na^{+}$	4023.76	4024.75				7.88	
	$+K^{+}$	4039.87	4040.62				13.72	
**Tetraicosasac-4Ac**	C162O110H265N5	4043.82	-	-	-	-	-	-
	$+Na^{+}$	4065.80	4066.23				8.00	
	$+K^{+}$	4081.91	4082.67				15.8	
**Pentaicosasac(25)-1Ac (5RU)**	C162O112H268N5	4078.84	-	-	-	-	-	-
	$+Na^{+}$	4100.82	4101.91				23.7	
	$+K^{+}$	4116.93	-				-	
**Pentaicosasac -2Ac**	C164O113H270N5	4120.88	-	-	-	-	-	-
	$+Na^{+}$	4142.86	4144.15				73.33	
	$+K^{+}$	4158.97	-				-	
**Pentaicosasac-3Ac**	C166O114H272N5	4162.92	-	-	-	-	-	-
	$+Na^{+}$	4184.90	4186.55				100	
	$+K^{+}$	4201.01	4201.95				15.84	
**Pentaicosasac -4Ac**	C168O115H274N5	4204.95	-	-	-	-	-	-
	$+Na^{+}$	4226.93	4228.19				93.7	
	$+K^{+}$	4243.04	4244.83				14.0	
**Pentaicosasac -5Ac**	C170O116H276N5	4246.99	-	-	-	-	-	-
	$+Na^{+}$	4268.97	4270.96				57.8	
	$+K^{+}$	4285.08	4286.14				9.75	
**Pentaicosasac-6Ac**	C172O117H278N5	4289.03	-	-	-	-	-	-
	$+Na^{+}$	4311.01	4312.83				21.8	
	$+K^{+}$	4327.12	-				-	
**Heptaicosasac(27)-2Ac 5RU+2Rha**	C176O121H291N5	4414.17	-	-	-	-	-	-
	$+Na^{+}$	4436.15	4437.55				8.85	
	$+K^{+}$	4452.26	-				-	
**Heptaicosasac -3Ac**	C178O122H293N5	4456.21	-	-	-	-	-	-
	$+Na^{+}$	4478.19	4478.92				12.6	
	$+K^{+}$	4494.30	-				-	
**Heptaicosasac -4Ac**	C180O123H295N5	4498.24	-	-	-	-	-	-
	$+Na^{+}$	4520.22	4521.30				12.3	
	$+K^{+}$	4536.33	-				-	
**Heptaicosasac-5Ac**	C182O124H297N5	4540.28	-	-	-	-	-	-
	$+Na^{+}$	4562.26	4563.11				7.99	
	$+K^{+}$	4578.37	-				-	
**Triakonsac (30)-2Ac (6RU)**	C196O135H324N6	4925.64	-	-	-	-	-	-
	$+Na^{+}$	4947.63	4948.51				7.99	
	$+K^{+}$	4963.73	-				-	

**Triakonsac -3Ac**	C198O136H326N6	4967.68	-	-	-	-	-	-
	+Na+	4989.66	4991.67				16.5	27.3
	+K+	5005.77	-				-	24.2
**Triakonsac -4Ac**	C200O137H328N6	5009.72	-	-	-	-	-	-
	+Na+	5031.70	5033.35				18.7	66.6
	+K+	5047.81	-				-	67.9
**Triakonsac -5Ac**	C202O138H330N6	5051.75	-	-	-	-	-	-
	+Na+	5073.74	5075.94				15.1	99.9
	+K+	5089.85	-				-	100
**Triakonsac -6Ac**	C204O139H332N6	5093.79	-	-	-	-	-	-
	+Na+	5115.77	5117.76				9.01	94.7
	+K+	5131.88	-				-	96.7
**Triakonsac -7Ac**	C206O140H334N6	5135.83	-	-	-	-	-	-
	+Na+	5157.81	5160.32					57.8
	+K+	5173.92	5176.66					65.4
**Triakonsac -8Ac**	C208O141H336N6	5177.87	-	-	-	-	-	-
	+Na+	5199.85	5202.13					21.6
	+K+	5215.96	5219.01					26.7
**Dotriakon sac(32)-4Ac 6RU+2Rha**	C212O145H347N6	5300.99	-	-	-	-	-	-
	+Na+	5322.97	-					-
	+K+	5339.08	5342.45					14.2
**Dotriakon sac-5Ac**	C214O146H349N6	5343.03	-	-	-	-	-	-
	+Na+	5365.01	5368.01					17.0
	+K+	5381.12	5384.94					19.5
**Dotriakon sac-6Ac**	C216O147H351N6	5385.07	-	-	-	-	-	-
	+Na+	5407.05	5410.98					14.0
	+K+	5423.16	5427.03					16.7
**Dotriakon sac-7Ac**	C218O148H353N6	5427.10	-	-	-	-	-	-
	+Na+	5449.09	-					-
	+K+	5465.19	5468.52					10.0
**Pentatriak onsac(35) -4Ac (7RU)**	C232O159H380N7	5812.47	-	-	-	-	-	-
	+Na+	5834.45	5838.89					21.8
	+K+	5850.56	5855.10					23.8
**Pentatriak onsac-5Ac**	C234O160H382N7	5854.51	-	-	-	-	-	-
	+Na+	5876.49	5880.90					40.2
	+K+	5892.60	5897.40					42.9
**Pentatriak onsac-6Ac**	C236O161H384N7	5896.54	-	-	-	-	-	-
	+Na+	5918.52	5923.05					47.6
	+K+	5934.63	5939.30					51.9
**Pentatriak onsac-7Ac**	C238O162H386N7	5938.58	-	-	-	-	-	-
	+Na+	5960.56	5965.13					36.7
	+K+	5976.67	5981.45					39.9
**Pentatriak onsac-8Ac**	C240O163H388N7	5980.62	-	-	-	-	-	-
	+Na+	6002.60	6007.02					18.1
	+K+	6018.71	6023.12					22.9
**Pentatriak onsac-9Ac**	C242O164H390N7	6022.65	-	-	-	-	-	-
	+Na+	6044.63	-					-
	+K+	6060.74	6065.63					10.3

Figure S7: Organic solvent stability of Sf6TSP

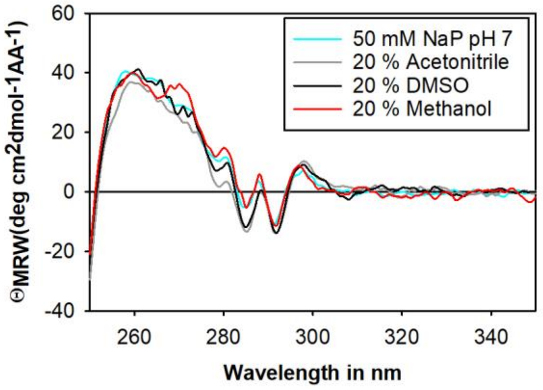

Figure S7. Organic solvent stability of Sf6TSP. Near-UV circular dichroism spectra monitored between 350-250 nm of 36 µM Sf6TSP in 50 mM sodium phosphate buffer pH 7 or with 20 % volume percent of organic solvent in the respective buffer.

Figure S8: Binding kinetics of Sf6TSP N340C labeled with NBD to SfY polysaccharide

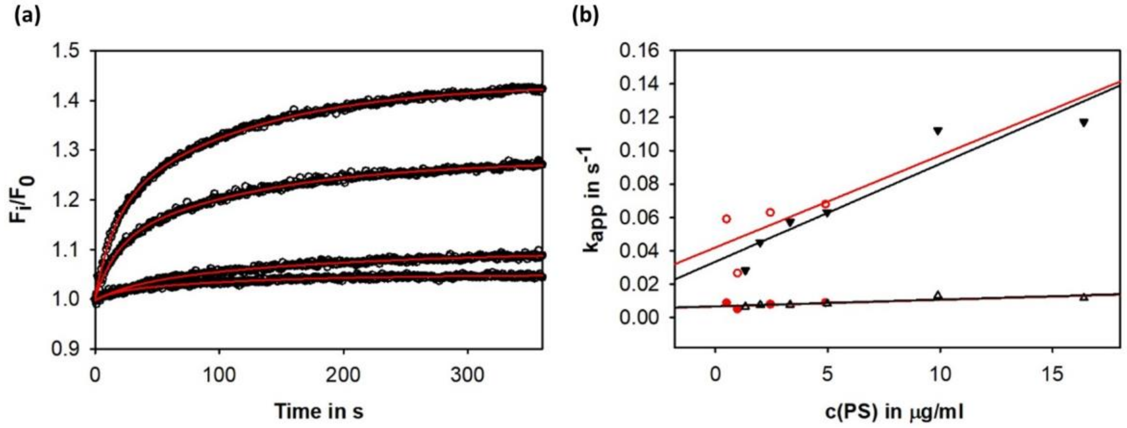

Figure S8. Binding kinetics of Sf6TSP N340C labeled with NBD to SfY polysaccharide. **(a)** Sf6TSP N340C-NBD (184 µM) excited at 492 nm and emission measured as kinetic traces of binding relaxations monitored at 540 nm between with the respective SfY polysaccharide concentrations in µg/ml were fitted by a biexponential equation, which resulted in k_{app}. **(b)** Rate constants from kinetic binding traces of Sf6TSP probed by intrinsic fluorescence excited at 280 nm (black) [1]. Rate constants from kinetic traces of Sf6TSP N340C NBD as shown in a (red). Dissociation rates were calculated after linear fitting by $k_{app}=k_{diss}+k_{ass}$ ([P]+[L]): k_{d1}(Sf6TSP N340C NBD)=0.0068/s, k_{d1}(Sf6TSP)=0.0059/s, k_{d2}(Sf6TSP N340C NBD)=0.0419/s, k_{d2}(Sf6TSP)=0.0335/s.

1. Kang, Y.; Gohlke, U.; Engström, O.; Hamark, C.; Scheidt, T.; Kunstmann, S.; Heinemann, U.; Widmalm, G.; Santer, M.; Barbirz, S. Bacteriophage Tailspikes and Bacterial O-Antigens as a Model System to Study Weak-Affinity Protein–Polysaccharide Interactions. *J. Am. Chem. Soc.* **2016**, *138*, 9109–9118, doi:10.1021/jacs.6b00240.

© 2018 by the authors. Submitted for possible open access publication under the terms and conditions of the Creative Commons Attribution (CC BY) license (http://creativecommons.org/licenses/by/4.0/).
