# Supplementary figures and images for: Bacteriophage Sf6 Tailspike Protein for Detection of Shigella flexneri Pathogens
Source: Viruses. 2018 Aug 15;10(8):431. doi: 10.3390/v10080431 (PMC6116271; doi:10.3390/v10080431)

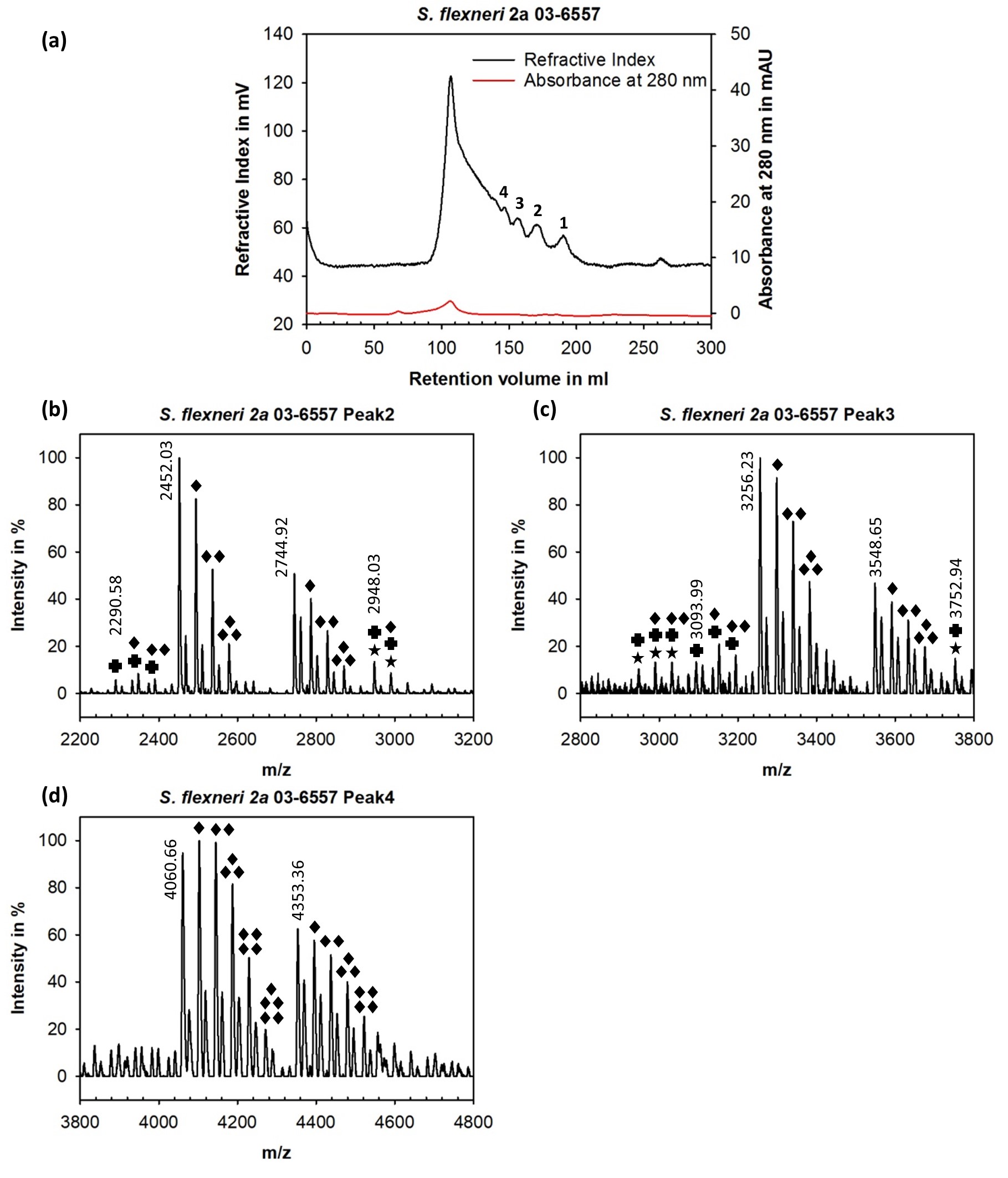

Supplement: Supplementary file 1 [file viruses-10-00431-s001.zip › MALDI_data_2a03-6557.jpg]

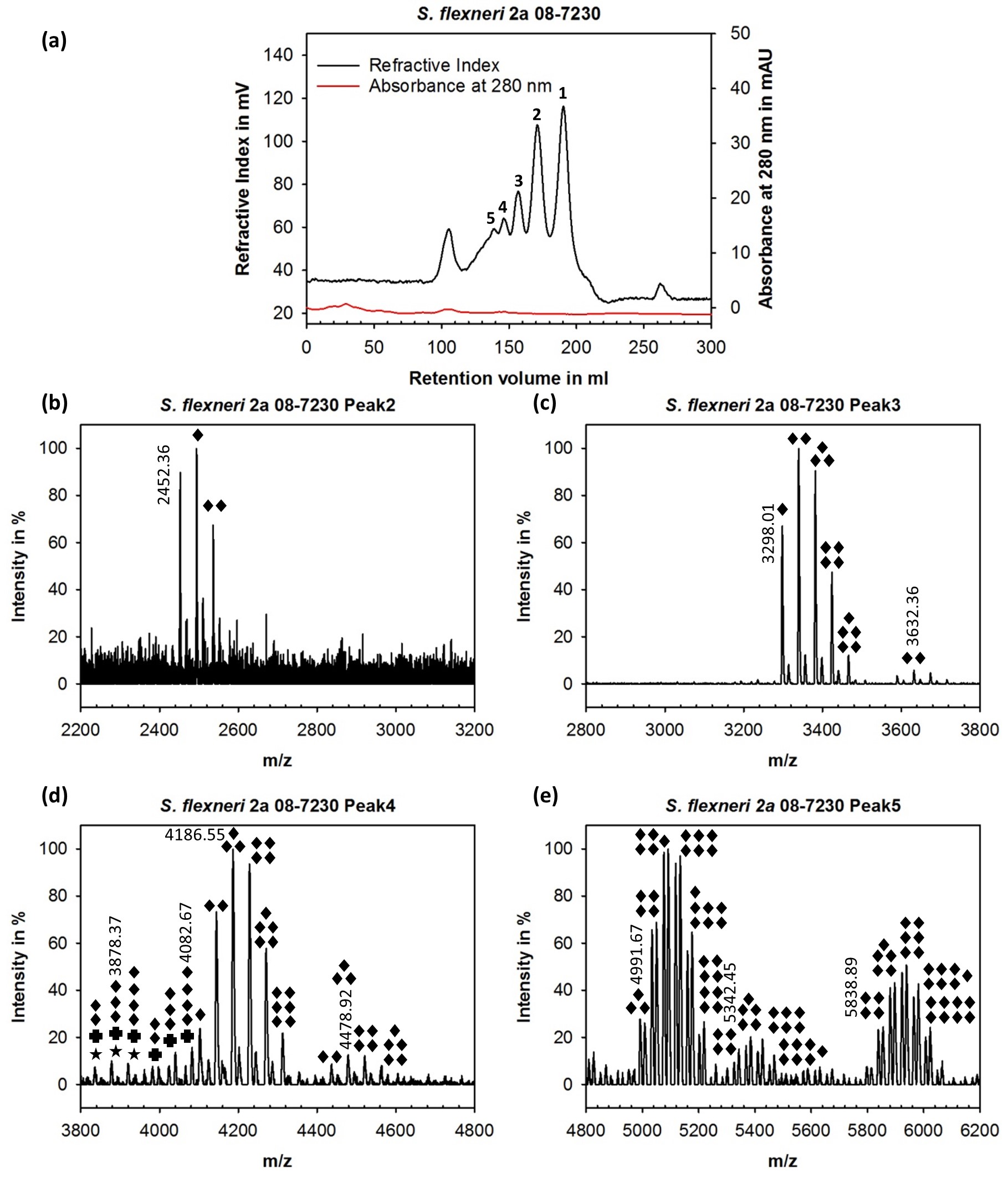

Supplement: Supplementary file 1 [file viruses-10-00431-s001.zip › MALDI_data_2a08-7230.jpg]

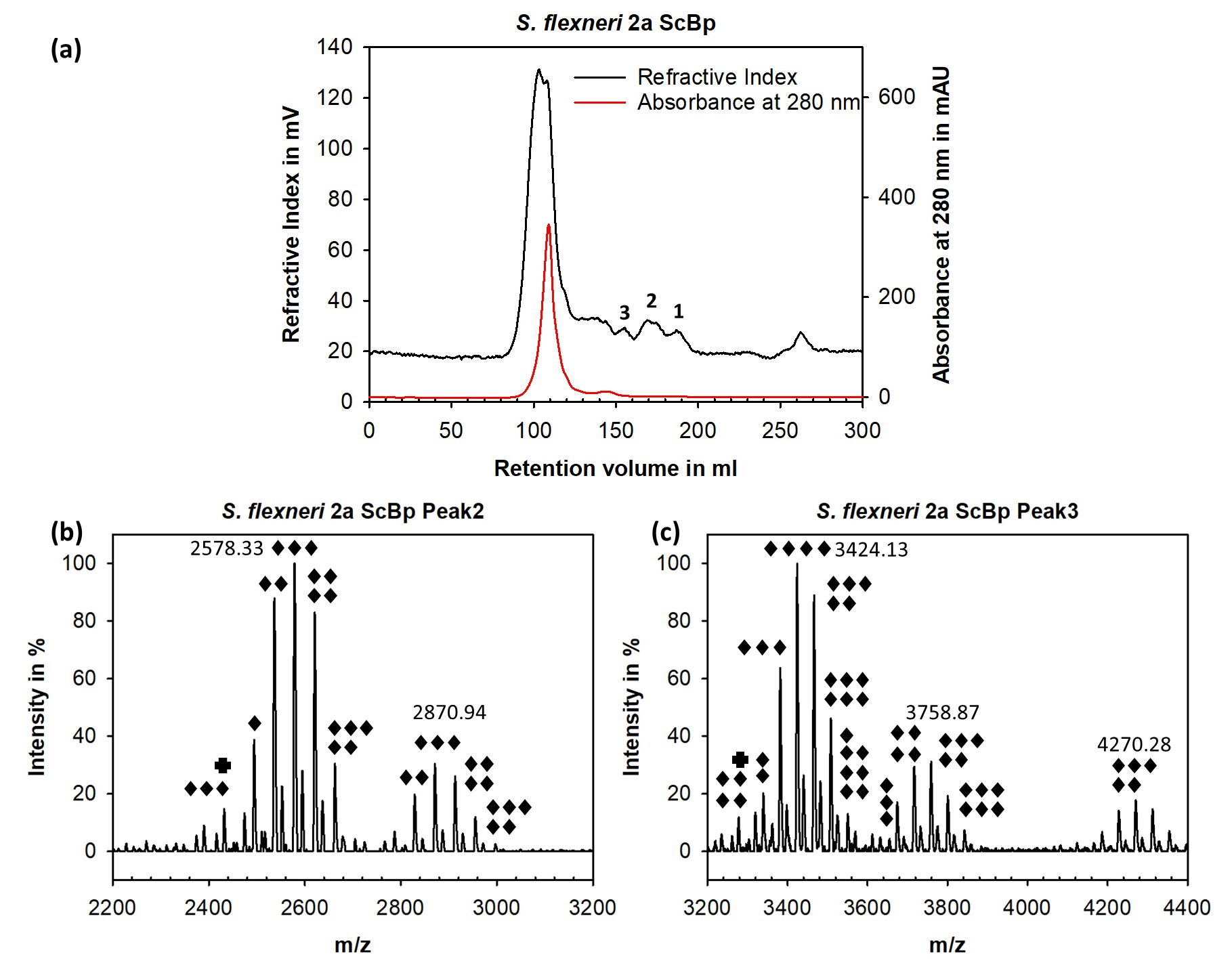

Supplement: Supplementary file 1 [file viruses-10-00431-s001.zip › MALDI_data_2aScan-Biopharma.jpg]

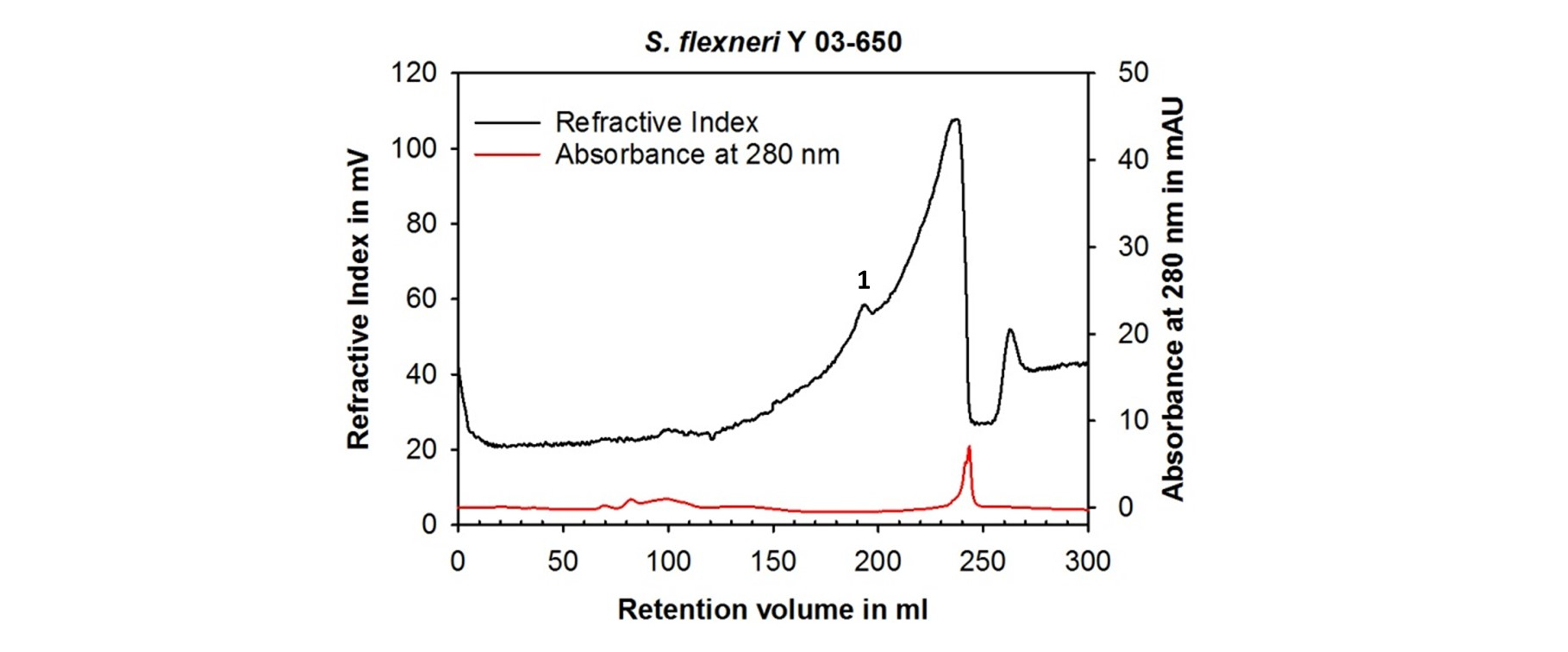

Supplement: Supplementary file 1 [file viruses-10-00431-s001.zip › MALDI_data_Y03-650.jpg]

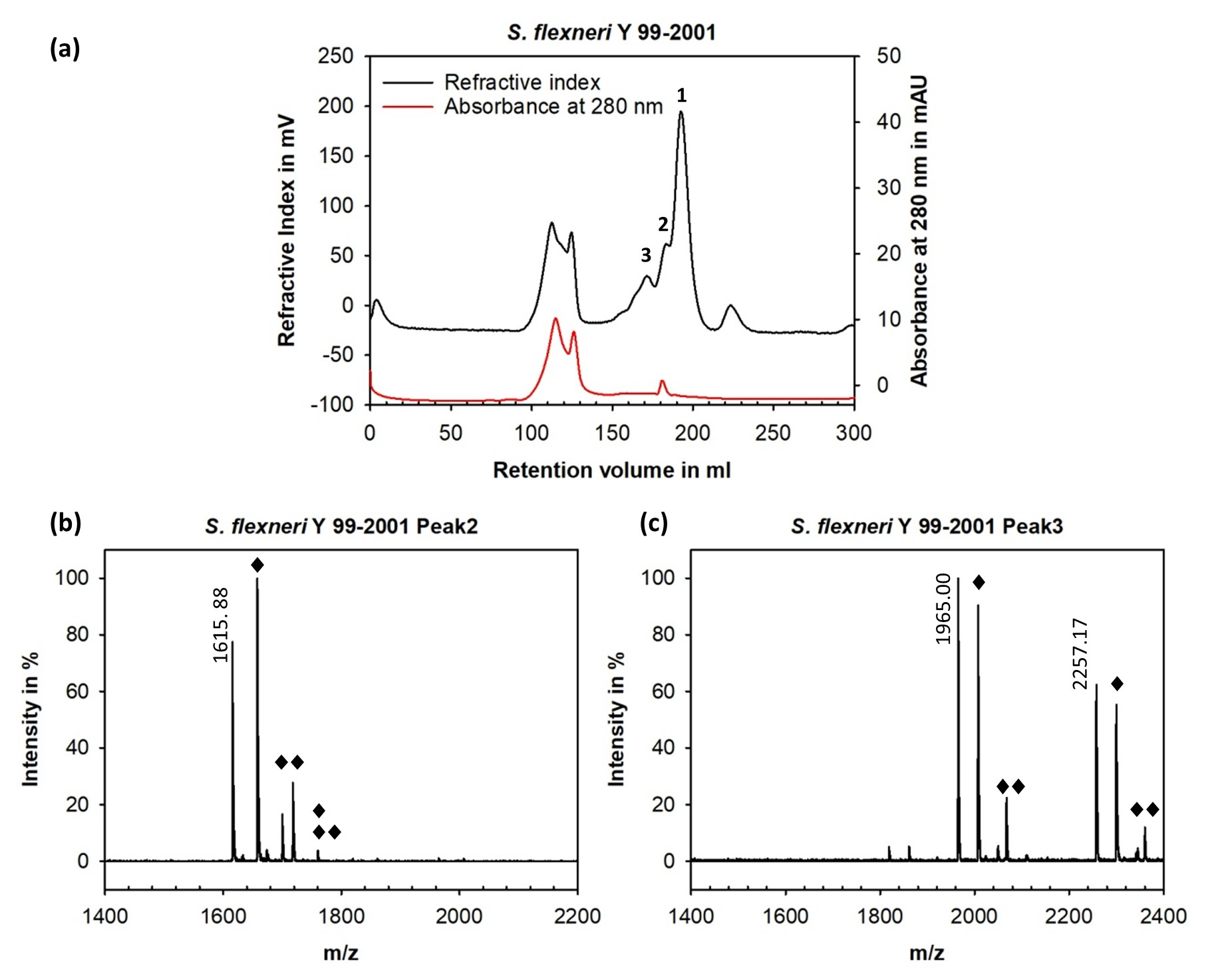

Supplement: Supplementary file 1 [file viruses-10-00431-s001.zip › MALDI_data_Y99-2001.jpg]

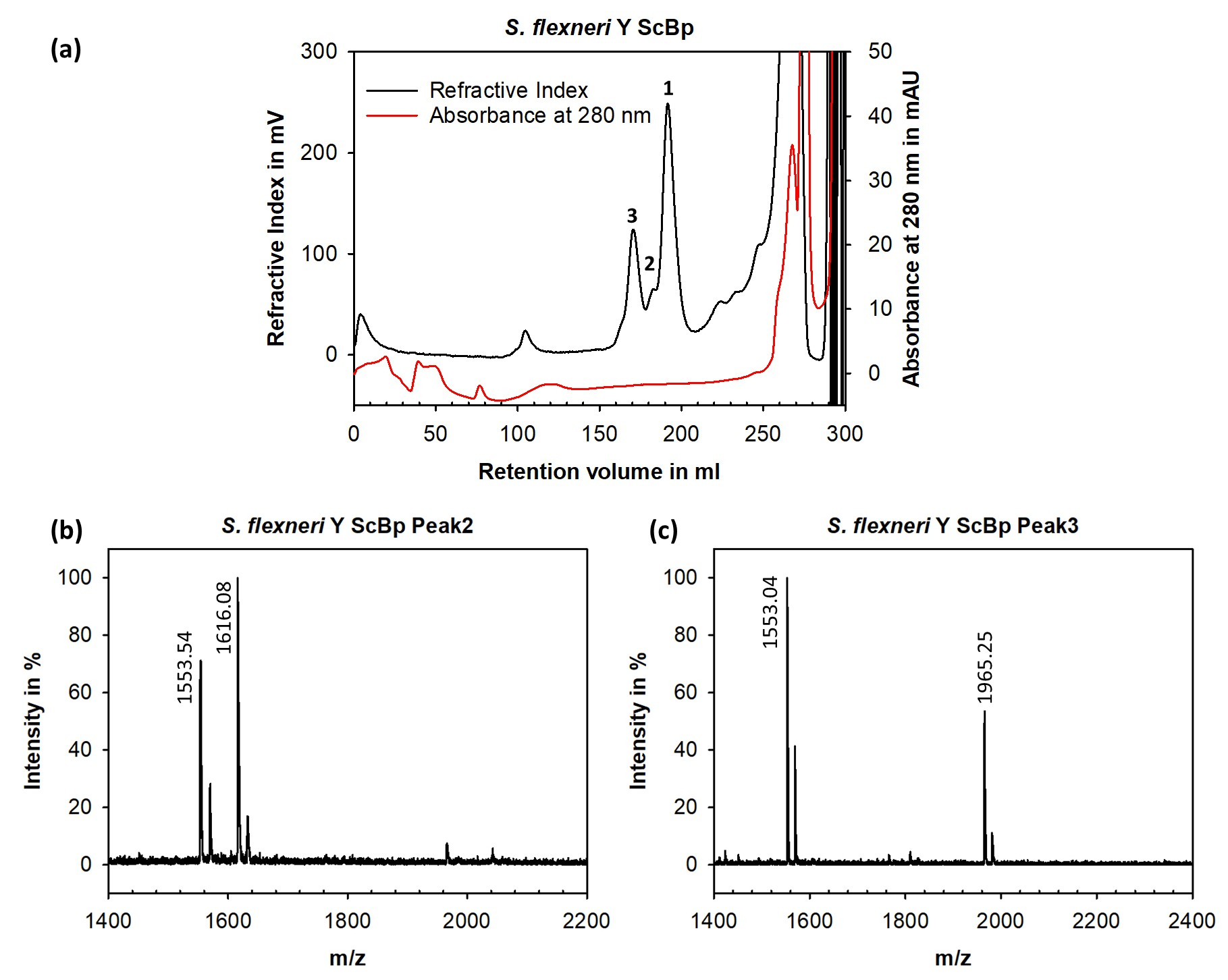

Supplement: Supplementary file 1 [file viruses-10-00431-s001.zip › MALDI_data_YScanBiopharma.jpg]

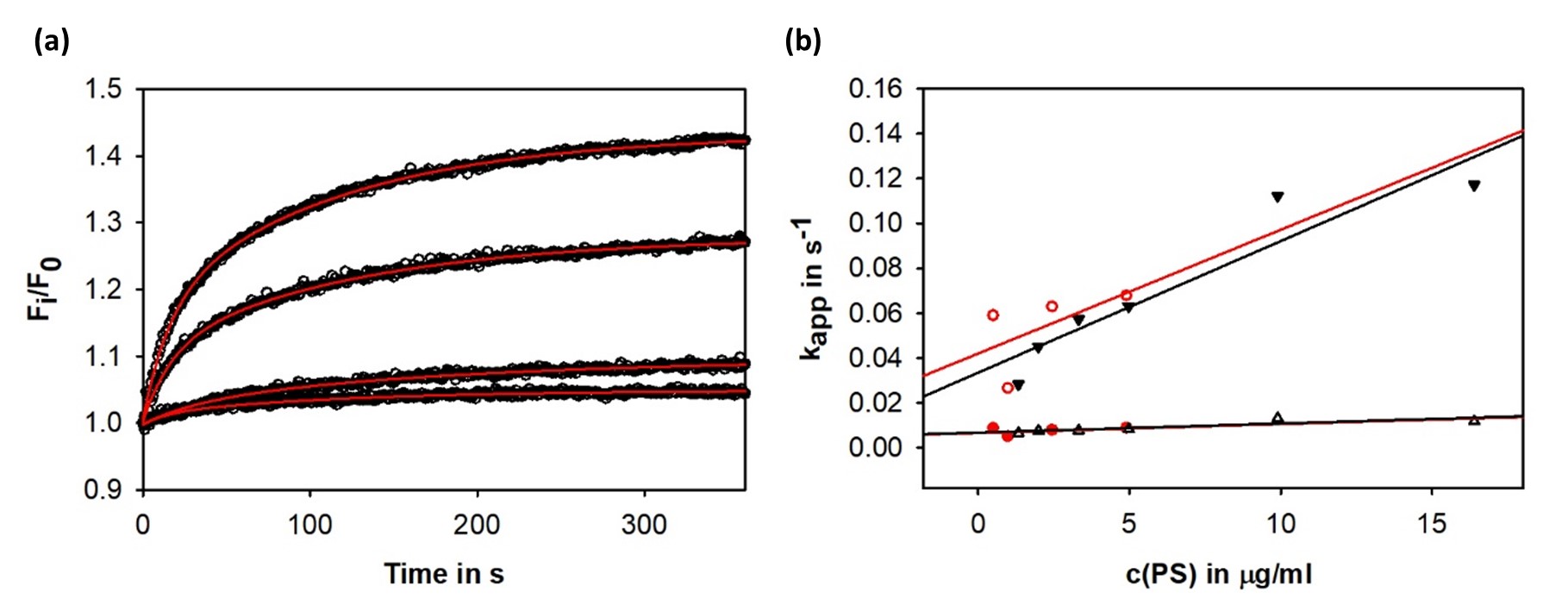

Supplement: Supplementary file 1 [file viruses-10-00431-s001.zip › N340C_kinetics_relaxations.JPG]

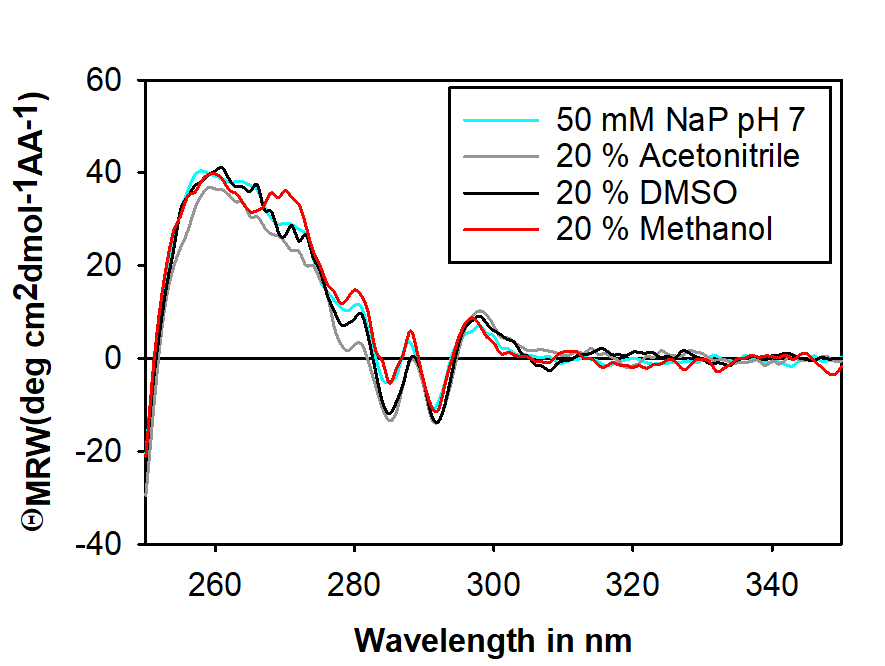

Supplement: Supplementary file 1 [file viruses-10-00431-s001.zip › Solvent_Stability.JPG]
